# Is behavioural activation an effective treatment for depression in children and adolescents? An updated systematic review and meta-analysis

**DOI:** 10.1007/s00787-024-02429-3

**Published:** 2024-04-14

**Authors:** Lucy Tindall, Philip Kerrigan, Jinshuo Li, Emily Hayward, Lina Gega

**Affiliations:** 1 Department of Health Sciences, University of York, Heslington, York YO10 5DD UK; 2 Hull York Medical School, University of York, Heslington, UK

**Keywords:** Psychological therapy, Mood disorders, Depression, Behavioural activation, Young people, Digital mental health, School interventions

## Abstract

Behavioural Activation (BA)—a brief therapy based on the scheduling of enjoyable, purposeful and rewarding activities—is an effective and cost-effective treatment for depression in adults that shows promise for children and adolescents. We provide an update on a previous systematic review of evidence on BA—delivered in-person, telephone, or online—for depression and comorbid anxiety in children and adolescents. We conducted systematic literature searches in 6 databases up to February 2024. We included all study designs evaluating BA with participants up to 18 years old with diagnosable depression, as established by a validated screening tool or diagnostic manual. We used the Moncrieff Scale and the Cochrane Risk of Bias tool to assess study quality. We summarised the findings of all study types with a narrative synthesis and of randomised controlled trials (RCTs) with a meta-analysis. Overall, 24 studies (6 RCTs, 18 pre-post evaluations, *n* = 2,758) met our inclusion criteria. A meta-analysis of 4 RCTs (*n* = 156) showed that BA has a small effect of 0.24 (Hedge’s adjusted g) in reducing depression symptoms compared to a waiting-list control, usual care and other therapies. Online and telephone-facilitated BA was shown to be feasible in 3 studies and effective in 1. Outcomes on comorbid anxiety were mixed. No economic evaluations met our inclusion criteria. BA shows sufficient promise as an intervention for reducing depression symptoms in children and adolescents to justify the need for further RCTs, providing that five conditions are met: studies are powered to detect a minimal clinically important difference; BA materials are fit-for-purpose to produce clinically meaningful change; follow-ups are longer than 6 months; primary outcomes are child-reported; and intervention costs, resource use and adverse events are reported.

**Supplementary Information:**

The online version contains supplementary material available at 10.1007/s00787-024-02429-3.

## Introduction

Depression is currently the fourth leading cause of illness and disability among adolescents aged 15–19 years and fifteenth for those aged 10–14 years globally [1]. Different treatment approaches, including watchful waiting, medication, cognitive behaviour therapy (CBT), interpersonal therapy (IPT) and non-directive supportive therapy (NDST), are recommended for young people with depression in the UK [2]. These are predominantly delivered through Child and Adolescent Mental Health Services (CAMHS); UK NHS-based services responsible for providing mental health support to children and young people. However, with increasing demand and limited resources, CAMHS have long waiting lists and high thresholds for accepted referrals [3] resulting in many young people with depression not receiving timely clinical interventions. Lack of, or delays in, treatment often lead to more severe presentations of depressive illness over time [4]. In response, more emphasis has been placed on expanding therapy provision into non-NHS services, including schools [5]. It is therefore opportune to examine options that may be effectively delivered across non-NHS services and by professionals not formally trained in providing mental health support.

One treatment that can be delivered within various settings and by professionals of different levels of expertise [6], including non-specialists outside clinical services [7–9], is Behavioural Activation (BA). The fundamental aim of BA is to increase positive reinforcement through restoring and increasing engagement in purposeful and rewarding activities. Through increased engagement it is hoped that there is a resulting positive emotional impact on an individual’s mood, relationships and energy [10]. This brief psychological treatment requires fewer sessions and shorter training than several more established therapies (e.g., CBT, IPT), making it a less-resource intensive alternative. Furthermore, BA’s focus upon withdrawal, inactivity, and avoidance, which are common symptoms in young people with depression [11], may make it better suited for this group.

The National Institute for Health and Care Excellence (NICE) in the UK recommends BA for adults with depression [12], but less is known about its delivery with young people. BA does not currently feature in any national [2] or international [13] recommendations for depression in young people. A 2017 systematic review examined the effectiveness of BA in the treatment of depression in young people [14]. The review included ten studies of which three were Randomised Controlled Trials (RCTs) and were pooled with a meta-analysis. The results demonstrated an effect in favour of BA over its comparators (CDRS-R: -4.2; 95% CI -8.25, -0.09). Whilst these findings suggested that BA may be effective in treating depression in young people, the paucity of studies highlighted the necessity of further research. A later meta-analysis [15] including four BA-focused RCTs, two of which were included in the meta-analysis by Tindall et al. [14] reported an effect in favour of BA vs. controls (1 active intervention, 1 signposting and 2 no treatment) with a pooled standardised mean difference of -0.7 (95% CI -1.20 to -0.20).

Since we completed our literature search in 2015 for our original review [14], increased focus has been placed on BA for young people. In 2021, the first known European feasibility RCT of BA for young people with depression [16] was conducted. The acceptability of BA and its promising outcomes when delivered in CAMHS or in school settings were shown in case reports [17–19] and pilot and feasibility studies [20, 21].

To broaden the provision of mental health interventions in the community, online delivery can be used to increase anonymity [22] and accessibility [23] and to reduce stigmatisation [24]. While Tindall et al. [14] did not identify any online versions of BA, the general shift to online therapy was expedited by the COVID-19 pandemic, urging further examination in this area.

## Aims and objectives

As recent years have seen an increased number of studies on BA with children and young people, we updated our earlier review by Tindall et al. [14] and we have included additional searches for economic evidence and for remote delivery by phone or online. The current review summarises the most up-to-date study-level evidence to answer four questions: (1) Is BA effective in treating young people with depression? (2) Does BA for depression improve comorbid symptoms of anxiety and quality of life? (3) Is BA for young people with depression cost-effective? (4) Can BA be delivered online or by telephone rather than in-person?

## Methods

We registered the review protocol on the International Prospective Register of Systematic Reviews (PROSPERO) (reference: CRD42023410806) and followed the Preferred Reporting Items for Systematic Reviews and Meta-Analyses (PRISMA) statement [25] to guide our methods.

### Information sources and screening

In March 2023 and February 2024, the following electronic databases were searched: Cochrane Library, EMBASE, MEDLINE, CINAHL Ultimate (EBSCO), PsychINFO, Scopus, and the ISRCTN registry. To cover peer review and grey literature sources, the Health Management Information Consortium, Open Grey, the Networked Digital Library of Theses and Dissertations and Web of Science Conference Proceedings were searched. The reference lists of all included studies were examined and reverse citation searching was completed in Google Scholar. We did not impose any restrictions on publication status or language.

All titles and abstracts identified were double screened by three reviewers (LT, PK, EH) against the pre-defined eligibility criteria. Where there was any uncertainty regarding a study’s inclusion, it was retained for full text screening. The same three reviewers conducted the full text screening with two reviewers independently screening each paper. Any disagreements were discussed until a consensus was reached. All screening was undertaken using Rayyan Software [26].

The search strategy (Supplementary Information S1) was based on three main constructs: *behavioural interventions* (i.e. behavioural activation, behavioural therapy, behavioural interventions, self-monitoring, activity scheduling), *depression* (i.e. depressive disorder, depressive, depression, depressed) and *young people* (i.e. adolescents, children, teen, youth, juvenile, pre-pubescent, student). The search period was set from 2015 to the present.

### Inclusion/exclusion criteria

Studies were included if at least 90% of their sample consisted of ≤ 18-year-olds with a diagnosis of depression, or with symptoms likely to be of diagnosable depression, as established by a validated screening tool or diagnostic manual. We included studies in which BA was a) based on a schedule of enjoyable, purposeful and rewarding activities, b) designed for depression c) offered as a standalone intervention, or as the core/dominant intervention, rather than as an equal part of a multicomponent intervention and d) delivered in any settings (e.g. schools, health services, community) and in any mode (e.g. face-to-face, online, by phone).

We included all types of quantitative study designs: RCTs, observational studies, pre-post evaluations and case studies. The primary outcome was depression/depressive symptoms measured by validated instruments, including self-report questionnaires and clinician/researcher administered measures. We were also interested in comorbid anxiety symptoms (measured by validated assessments), cost-effectiveness data and quality of life (QoL) outcomes. No restrictions were placed on the length of follow-up (when outcomes were measured) but we only included studies with at least two assessment points, one of which was at baseline.

### Data extraction

Three reviewers used a data extraction proforma, which was first piloted for consistency with three papers, to record the following information as reported in the included studies: study characteristics (study name, author(s), year of publication/production, location, and setting), study design, study populations (basic participant demographics, depression diagnosis methods), intervention and comparator details (intervention/comparator type, duration, session number), and relevant outcome data for effect size calculations (depression severity, unit of measurement).

### Quality assessments

The original review [14] assessed the quality of all included studies using the Moncrieff Scale [27] plus the Cochrane Risk of Bias (RoB) tool [28] for the RCTs only. In this review, we used the Cochrane RoB tool (newest version, RoB-2) [29] to assess the quality of the included RCTs. Based on this tool, RCTs were graded in terms of their ‘bias’ as either ‘low risk, ‘some concerns’ or ‘high risk’. We used the Moncrieff Scale to assess the quality of both RCTs and pre-post evaluations, by attributing a score of 0, 1 or 2 to 23 risk items, with higher scores denoting higher study quality.

### Data synthesis

We carried out a narrative synthesis of the results of all 24 studies and a meta-analysis of RCTs that reported outcomes based on the Children’s Depression Rating Scale – Revised (CDRS-R) [30], using a random-effects model and displaying the results in forest plots. All analyses were undertaken in Stata version 18 [31].

Two RCTs [16, 32] reported complete cases whilst four reported imputed results [23, 33–35]. For our analyses, we included the primary results as reported by authors, irrespective of whether the results were based on complete cases or imputed outcomes. We defined our primary outcome as the data reported by a study at end of treatment or at the earliest follow-up point, although this may not have necessarily been the primary follow-up point for the study. Where studies had three treatment arms, and one of these was BA, this was taken as the intervention and the other treatment arm that was a placebo/waiting list (rather than another active treatment) was selected as the comparator.

In one study [34] two forms of BA were compared with usual care. To address this within the meta-analysis, and in alignment with the Cochrane handbook for systematic reviews of interventions recommendations (version 6.3) [36], the pooled mean and standard deviation (SD) were calculated based on the two intervention groups and formed a single BA group. The meta-analysis included only self-reported outcomes by young people and not parent-reported outcomes.

Statistical heterogeneity was assessed using the *I*^*2*^ statistic with a value of 25% regarded as low, 50% as moderate, and 75% as high [37]. Publication bias was assessed using funnel plots.

## Results

### Retrieved and selected studies

We conducted our original searches in July and August 2015, identifying 5,931 records, of which 5,495 were screened, after removing duplicates (*n* = 436). Title and abstract screening identified 42 full-text articles of which ten were eligible for inclusion. We updated the same searches in March 2023 and February 2024 and identified 25,414 records, of which 17,024 were screened, after removing duplicates (*n* = 8,390). Reverse citation searching identified one additional eligible paper [38] that had not been identified in the original review.

Title and abstract screening identified 62 articles for full-text review, of which 14 were eligible and 48 were excluded for the following reasons: the sample was predominantly over 18 year olds, (more 90% aged ≤ 18 years) (*n* = 27), the sample included participants without depression at baseline (*n* = 11), the intervention was mixed and not standalone BA (n = 5), the BA was not developed for depression (*n* = 1), depression was not included as an outcome (*n* = 1), the inclusion criteria were not specified (*n* = 1), pre/post data was not included/was missing (*n* = 1), the reporting paper was inaccessible (*n* = 1). (Reasons for exclusion can be seen in Supplementary Information S2). Where necessary, authors were contacted to request additional information during data extraction.

**Fig. 1:**
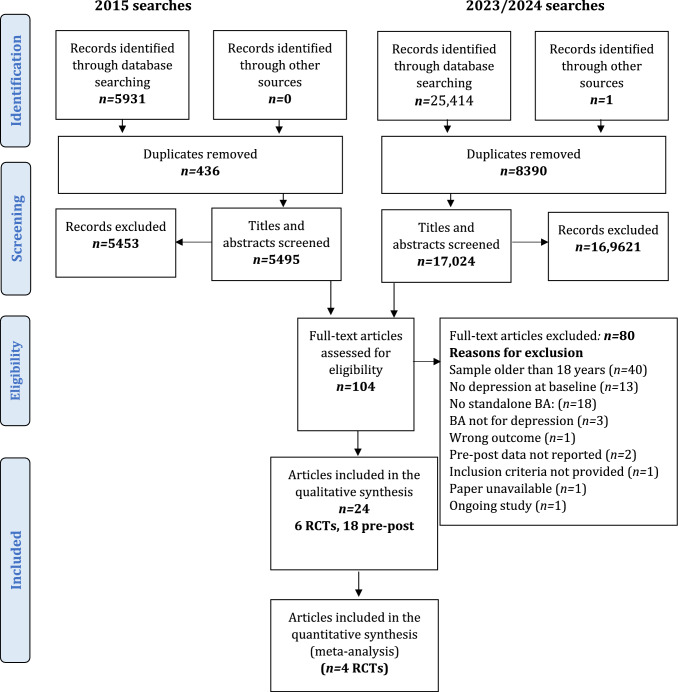
PRISMA Diagram. From: Moher D, Liberati A, Tetzlaff J, Altman DG, The PRISMA Group (2009). Preferred Reporting Items for Systematic Reviews and Meta-Analyses: The PRISMA Statement. PLoS Med 6(7): e1000097. doi:0.1371/journal.pmed100009.

The PRISMA diagram of the updated searches is presented in Fig. 1, the PRISMA of the earlier review can be found in Tindall et al. [14].

### Characteristics of included studies

#### Study design

A total of 24 studies, published between 1985 and 2024, met our inclusion criteria. Six (6) were RCTs [16, 23, 32–35] and 18 were pre-post evaluations [17, 18, 38–53] in which outcomes were measured at baseline and at a minimum one follow-up point. The pre-post-evaluations included single case studies, one-group within-participant designs and multiple non-randomised groups.

#### Sample

Participants were between 8 and 18 years old. The largest study by Schleider et al. [23] included 2,452 participants, whereas the total sample size across the remaining 23 studies was 306 participants, ranging from single case studies to 60 participants [35]. Most studies (*n* = 15) included a higher proportion (> 50%) of girls/young women (*n* = 8) or a female-only sample (*n* = 7), and only 5 studies had a gender balance or included more boys/young men.

#### Study settings

More than half of the studies (*n* = 14) took place in the USA [23, 32, 33, 35, 38, 40, 42, 43, 45–49, 52], and the rest we carried out in the UK (*n* = 7) [16–18, 39, 42, 44, 53], Australia [51], Sweden [34] and the United Arab Emirates [50]. BA was delivered in clinics/treatment centres (*n* = 15), schools (*n* = 8) and the community (*n* = 1).

#### Interventions and comparators

In 20 of 24 studies BA was delivered in-person by professionals including clinical psychologists, students (graduate, doctoral and post-doctoral), mental health clinic staff, social workers, school counsellors, psychological wellbeing practitioners and study therapists. BA was delivered online in two studies [23, 34] and via videocall in two studies [41, 53], one of these due to the COVID-19 pandemic.

The number of BA sessions ranged from 1 to 22, with a typical frequency and duration of weekly sessions lasting 20–30 min or 1 h. Most studies delivered BA in one-to-one sessions, although two studies [33, 45] were group BA. Twenty (20) studies followed a standardised treatment manual for professionals, and three of those [17, 41, 51] also used a workbook for young people.

Comparators in RCTs included a waiting list, usual care (e.g., CBT, medication, Psychodynamic Therapy, IPT) and/or alternative psychological and supportive therapies (e.g., Self-Control Therapy, Supportive-Control Therapy, Growth Mindset Single Session Intervention [GM-SSI]) or a psychological placebo.

#### Outcome measures

CDRS-R [30] and the Kiddie Schedule for Affective Disorders and Schizophrenia (K-SADS) [54] were the most frequently used depression outcome measures. Eight (8) studies also assessed anxiety. Only two studies [34, 42] reported QoL data using the Family Quality of Life Scale (FQOLS)and KIDSCREEN-10 index, respectively. The outcome measures are detailed in Table 1.

**Table 1:** Characteristics of included studies by study design

Randomised controlled trials				
Study	Setting and sample	Intervention	Comparator	Outcome Measures
Chu et al. (2016) [33]	**Setting:** One public middle school, USA **Participants** *n* = 35 **Age:** 12–14 (*M*:12.03, *SD*:0.45) **Gender:** 10 males (29%), 25 females (71%) **Diagnosis:** Current clinical principal diagnosis of either a unipolar depression disorder or an anxiety disorder based on CDRS-R or ADIS-IV (no cut-offs specified)	Group behavioural activation therapy (GBAT) **Duration:** 10 1-h sessions over 10 weeks **Delivery:** Group-based, face-to-face (1 clinical psychologist, 4 graduate students, 2 school counsellors) ***n*** = 21 **Manual:** Yes	Waiting list ***n*** = 14	**Depression measures:** CDRS-R CES-D-P CES-D-C **Anxiety measures:** ADIS-IV SCARED-C SCARED-P **QoL measures:** N/A **Timings:** Pre-treatment Post-treatment 4-months follow-up
Grudin et al. (2022) [34]	**Setting:** Specialist Outpatient Clinic, Sweden **Participants**: *n* = 32 **Age:** 13–17 (*M*: 15.4, *SD*:1.6) **Gender:** 13 males (41%), 19 females (59%) **Diagnosis:** Diagnosis of mild or moderate MDD according to the DSM-5 Criteria	Therapist-guided internet-delivered BA **Duration:** 8 sessions (‘chapters’) lasting 30–60 min delivered over 10 weeks **Delivery:** Individuals, online, completed with support from a clinical psychologist ***n*** = 11 **Manual:** No Self-guided internet-delivered BA **Duration:** 8 sessions (‘chapters’) lasting 30–60 min delivered over 10 weeks **Delivery:** Individual, online, completed independently ***n*** = 10 **Manual:** No	Usual care (i.e., referral to usual care within child and youth psychiatry or primary care. Treatments included medication, CBT, supportive therapy, Psychodynamic therapy) **Duration:** Varied – 0 to 10 sessions **Delivery:** Varied and included: pharmacological interventions, psychological interventions, supportive interventions or a combination of these, as well as psychiatric or neuropsychiatric assessments and medications ***n*** = 11	**Depression measures:** MINI-KID CDRS-R SMFQ–C SMFQ-P **Depression and anxiety measures:** RCADS **QoL measures:** KIDSCREEN 10 Index **Timings:** Pre-treatment Post-treatment 3-months follow-up
Kitchen et al. (2021) [16]	**Setting:** Three Child and Adolescent Mental Health Services (CAMHS), UK **Participants:** *n* = 22 **Ages:** 12–17 (*M*:15.7, *SD*:1.2) **Gender:** 4 males (18%), 18 females (82%) **Diagnosis:** DSM diagnosis of MDD according to the Kiddie-SADS-Present and Lifetime (K-SADS-PL) version	Behavioural Activation **Duration:** 8 sessions lasting one hour and delivered weekly **Delivery:** Individual, face-to-face (7 existing CAMHS staff NHS band 4–7) ***n*** = 11 **Manual:** Yes	Usual Care (standard care in CAMHS as delivered appropriate by CAMHS professionals, no restrictions placed. Treatments includes CBT, information sessions, coping skills support) **Duration:** Variable depending on therapy, ranging from 0 to 9 sessions with varied timings **Delivery:** Delivered by professionals not trained in BA ***n*** = 11 **Manual:** No	**Depression measures:** K-SADS MFQ-C MFQ-P **Anxiety measures:** N/A **QoL measures:** N/A **Timings:** 3-month follow-up (K-SADS) 6-month follow-up (MFQ)
McCauley et al. (2015) [35]	**Setting:** One hospital-based mental health clinic, USA **Participants*:**** n* = 60 **Age:** 12 to 18 (*M*: 14.9, *SD*:1.53) **Gender:** 22 males (37%), 38 females (63%) **Diagnosis:** Depressive disorder based on K-SADS diagnostic interview	Adolescent behavioural activation program (A-BAP) **Duration:** 14 sessions **Delivery:** Individual, face-to-face (2 doctoral students, 1 social worker) ***n*** = 35 **Manual:** Yes	Evidence-based practice for depression (EBP-D) (Treatments included CBT and IPT) **Duration:** ≤ 14 sessions **Delivery:** Face-to-Face ***n*** = 25	**Depression measures:** K-SADS diagnostic interview CDRS-R SMFQ **Anxiety measures:** MASC **QoL measures:** N/A **Timings:** Pre-treatment Post-treatment 6-months follow-up 12-months follow-up
Schleider et al. (2022) [23]	**Setting:** Nationwide, Community-based, USA **Participants:** *n* = 2,452 **Age:** 13–16 **Gender:** 251 males (10%), 2160 females (88%), 21 other (1%), 20 prefer not to say (1%) **Diagnosis:** Screened positive for MDD based on the PHQ-2 screener	Behavioural Activation Single Session Intervention (BA-SSI) **Duration:** One session lasting 20–30 min **Delivery:** Individual, online, self-guided ***n*** = 821 **Manual:** No	Growth Mindset Single Session Intervention (GM-SSI) **Duration:** One session lasting 20–30 min **Delivery:** Individual, online, self-guided ***n*** = 813 **Manual:** No Supportive Control **Duration:** One session lasting 20–30 min **Delivery:** Individual, online, self-guided ***n*** = 818 **Manual:** No	**Depression measures:** CDI-SF **Anxiety measures:** GAD7 **QoL measures:** N/A **Timings:** Pre-treatment 3-months follow-up
Stark (1985) [32]	**Setting:** One elementary school, USA **Participants**: *n* = 29 **Age:** 9–12 years (*M*: 11.2) **Gender:** 16 males (55%), 13 females (45%) **Diagnosis:** Depressive disorder based on CDI (≥ 16)	Behaviour therapy **Duration:** 12 45-min sessions over 5 weeks **Delivery:** Group-based, face-to-face (1 study therapist, 1 clinical psychologist) ***n*** = 10 **Manual:** Yes	Self-Control Therapy: **Duration:**12 45-min sessions over 5 weeks **Delivery:** Face-to-Face ***n* = **9 Waiting list **Duration:** N/A **Delivery:** N/A ***n* = **9	**Depression measures:** CDI CDS CDRS-R **Anxiety measures:** RCMAS **QoL measures:** N/A **Timings:** Pre-treatment Post-treatment 8-weeks follow-up
Pre-Post Evaluations				
Arnott et al (2020) [39]	**Setting:** One secondary school, UK **Participants:** *n* = 8 **Age:** 12–15 (*M*:14.27; *SD*:0.97) **Gender:** 4 males (50%), 4 females (50%) **Diagnosis:** Met diagnostic criteria for MDD or depressive disorder not otherwise specified by a consultant adolescent psychiatrist, measured using PHQ-2	Behavioural activation **Duration:** 8–12 weekly 1-h sessions **Delivery:** Individual face-to-face (graduate therapist) ***n*** = 4 **Manual:** Yes	Waiting list **Duration:** N/A **Delivery:** N/A ***n*** = 4	**Depression measures:** K-SADS-PL SMFQ-C **Anxiety measures:** N/A **QoL measures:** N/A **Timings:** Pre-treatment Post-treatment 4-months follow-up
Brett et al (2020) [44]	**Setting:** Secondary schools, UK **Participants: *n* = **2 **Age:** 16 (male) & 15 (female) **Gender:** 1 male (50%), 1 female (50%) **Diagnosis:** Primary presentation of depression or sub-clinical symptoms of depression	Brief Behavioural activation (Brief BA) **Duration:** 6–8 sessions of 40–50 min **Delivery:** Individual face-to-face (qualified psychological wellbeing practitioner) ***n*** = 2 **Manual:** Yes	N/A	**Depression measures:** K-SADS-PL (depression section only) RCADS depression subscale **Anxiety measures:** N/A **QoL measures:** N/A **Timings:** Pre-treatment (K-SADS-PL, RCADS) After each session (RCADS only) Follow-up at one month (RCADS only)
Chu et al. (2009) [45]	**Setting:** One public middle school, USA **Participants:** *n* = 5 **Ages:** 12–14 **Gender:** 2 males (40%), 3 females (60%) **Diagnosis:** Current clinical principal diagnosis of either a unipolar depression disorder or an anxiety disorder based on CES-D (≥ 15) or ADIS-IV (no cut-offs specified)	Group behavioural activation therapy (GBAT) **Duration:** 13 sessions over 13 weeks **Delivery:** Group-based, face-to-face (mental health specialists) ***n*** = 5 **Manual:** Yes	N/A	**Depression measures:** CES-D-C CES-D-P **Anxiety measures:** ADIS-IV CSR MASC-C MASC-P **QoL measures:** N/A **Timings:** Pre-treatment Post-treatment (13 weeks)
Douleh (2013) [40]	**Setting:** 2 High schools, USA **Participants*:**** n* = 14 **Age:** 14 – 18 (*M*: 15.71) **Gender:** 8 males (57%), 6 females (43%) **Diagnosis:** Depressive disorder based on CDRS-R (≥ 45)	Motivational Interviewing (MI) **Duration:** 1 to 4 sessions over 4 weeks **Delivery:** Face-to-face (study therapist) ***n*** = 14 MI and Fun activities (FA) **Duration:** 1 to 4 sessions over 4 weeks **Delivery:** Individual, face-to-face (study therapist) ***n*** = 7 MI and FA and values based behavioural activation (VBBA) **Duration:** 1 to 4 sessions over 6 weeks **Delivery:** Individual, face-to-face ***n*** = 1 **Manual:** Yes	N/A	**Depression measures:** CDRS-R BDI-II MINI-KID **Anxiety measures:** N/A **QoL measures:** HRQOL (measure not specified) **Timings:** A1: Pre-treatment A2: Post-MI (4 weeks post pre-treatment) A3: Post-FA (10 weeks post pre-treatment) A4: Post-VBBA (16 weeks post pre-treatment) A5: 20 weeks post treatment
Dubicka et al. (2022) [41]	**Setting:** Specialist child and adolescent mental health service (CAMHS), UK **Participants:** *n* = 36 **Age:** 12–17 (*M*: 14.5; *SD*: 1.2) **Gender:** 12 males (33%), 24 females (66%) **Diagnosis**: Individuals referred to CAMHS with and scored ≥ 27on the MFQ	Behavioural Activation **Duration:** 8 sessions lasting 45 min (flexible depending on need) **Delivery:** Individual, face-to-face or remotely (video, telephone or text) during the COVID-19 **Duration:** ***n*** = 33 **Manual:** Yes (as well as workbooks)	N/A	**Depression measures:** MFQ **Anxiety measures:** N/A **QoL measures:** **Timings:** Pre-treatment Post-treatment
Jacob et al. (2013) [46]	**Setting:** Community mental health clinics, USA **Participants:** *n* = 3 **Ages:** 14–17 **Gender:** 2 males (66%), 1 female (33%) **Diagnosis:** Depressive disorder based on K-SADS, CDRS-R (≥ 45) and BDI-II (≥ 14)	Behavioural Activation (adapted for low-income, African-American adolescents) **Duration:** 14–17 sessions over 6 months (length of sessions not specified) **Delivery:** Individual, face-to-face (3 study therapists) ***n*** = 3 **Manual:** Yes	N/A	**Depression measures:** K-SADS CDRS-R BDI-II **Anxiety measures:** N/A **QoL measures:** N/A **Timings:** Pre-treatment At each session (BDI-II) Week 9 (CDRS-R) Post-treatment (6 months)
Jenness et al. (2023) [47]	**Setting:** Clinical Centre, USA **Participants:** *n* = 1 **Ages:** 14 **Gender:** Female **Diagnosis:** Depression diagnosis determined by K-SADS and clinical interviews with the parents & child together and separately conducted by the lab's psychiatrist and clinical social worker	Adolescent Behavioural Activation Program **Duration:** 13 sessions delivered weekly **Delivery:** Individual, face-to-face (clinical psychologist) ***n* = **1 **Manual:** Yes	N/A	**Depression measures:** SMFQ **Anxiety measures:** SCARED LSAS **QoL measures:** N/A **Timings:** Pre-treatment End of treatment 12-months
Mohamed et al. (2024) [53]	**Setting: C**hild and adolescent mental health service (CAMHS), UK **Participants:** *n* = 7 **Ages:** 13–17 **Gender** 5 males (71%) and 2 females (29%) **Diagnosis:** Primary presentation of depression (a diagnosis of depression or moderate-severe symptoms of depression) in the context of a clinically confirmed autism diagnosis	**Duration:** 12 sessions lasting c. 1 h delivered weekly **Delivery:** Individual, video-call with trainee Clinical Psychologist, registered Clinical Psychologist, or post-doctoral research psychologist under supervision of a registered Clinical Psychologist ***n* = **6 **Manual:** Yes	N/A	**Depression measures:** BDI-II RCADS-C RCADS-P **Anxiety measures:** RCADS-C RCADS-P **QoL measures:** N/A **Timings:** Pre-treatment Mid-treatment (not reported) End of treatment
Nabors, Klein and Graves (2021) [48]	**Setting:** School-based, USA **Participants:** *n* = 1 **Ages:** 13 **Gender:** Female **Diagnosis:** Depressive symptoms measured by the CDRS-R	**Duration:** 12 sessions, delivered weekly, sessions 1 to 6 lasted 45 min and sessions 7 to 12 lasted 15 to 30 min **Delivery:** Individual, face-to-face sessions delivered by a school psychologist ***n* = **1 **Manual:** No	N/A	**Depression measures:** CDRS-R PROMIS depression scale **Anxiety measures:** N/A **QoL measures:** N/A **Timings:** *CDRS-R:* Pre-treatment One-month post-treatment start Two-months post-treatment start End of treatment *PROMIS Depression scale:* Biweekly intervals
Pass, Hodgson, et al. (2018) [17]	**Setting:** One Child and Adolescent Mental Health Service (CAMHS), UK **Participants:** *n* = 1 **Age:** 16 **Gender:** Female **Diagnosis:** Clinical range for depression, panic, and separation anxiety assessed by diagnostic interview and RCADS	Brief Behavioural Activation for Adolescent Depression **Duration:** 8 one-hour sessions delivered weekly **Delivery**: Individual, face-to-face (psychology graduate) ***n* = **1 **Manual:** Yes (as well as workbooks)	N/A	**Depression measures:** RCADS depression subscale- C RCADS depression subscale—P **Anxiety and depression measures:** RCADS-C RCADS-P **QoL measures:** N/A **Timings:** Pre-treatment End of treatment RCADS depression subscale: completed at each session
Pass et al. (2016) [18]	**Setting:** Child and Adolescent Mental Health Service (CAMHS), UK **Participants:** *n* = 1 **Age:** 16 **Gender:** Female **Diagnosis:** Moderate MDD assessed by the K-SADS	Brief Behavioural Activation for Adolescent Depression **Duration:** 9 one-hour sessions delivered weekly **Delivery**: Individual, face-to-face (clinical psychologist) ***n* = **1 **Manual:** Yes	N/A	**Depression measures:** K-SADS RCADS depression subscale-P RCADS depression subscale-C **Anxiety measures:** N/A **QoL measures:** N/A **Timings:** K-SADS: Pre-treatment RCADS: Pre-treatment Session 8 6-weeks follow-up RCADS depression subscale: completed at each session
Riley & Gaynor (2014) [42]	**Setting:** 3 elementary schools, 1 middle school, USA **Participants:** *n* = 11 **Age:** 8–12 years (*M*: 9.8, *SD*: 1.26) **Gender:** 9 males (82%), 2 females (18%) **Diagnosis:** Depressive disorder based on CDRS-R (≥ 12) and CDI (≥ 40)	Non-directive therapy (NDT) only: **Duration:** 3 sessions over 3 weeks **Delivery:** Individual, face-to-face (Doctoral students) ***n*** = 11 NDT and behaviour therapy (BT) As above plus 9 BT sessions **Manual:** Yes	N/A	**Depression measures:** CDRS-R CDI **Anxiety measures:** N/A **QoL measures:** FQOLS **Timings:** Pre-treatment Post-treatment (both groups) 2-months follow-up
Ritschel et al. (2016) [43]	**Setting:** Outpatient treatment clinic, USA **Participants:** *n* = 28 **Age:** 14–17 (*M*: 15.34) **Gender:** 9 males (22%), 19 females (68%) **Diagnosis:** MDD as a primary diagnosis based on the K-SADS and who had raw scores of ≥ 45 on the CDRSR	Behavioural Activation Duration: 22 sessions over 18 weeks Delivery: Individual, face-to-face (3 doctoral level psychologists, 2 advanced graduate students) ***n* = **22 **Manual:** Yes	N/A	**Depression measures:** K-SADS CDRS-R CBCL (withdrawn/depressed scale) BDI-II **Anxiety measures:** N/A **QoL measures:** N/A **Timings:** Pre-treatment Post-treatment Week 9 Week 18 BDI-II: completed fortnightly)
Ritschel et al. (2011) [49]	**Setting:** Outpatient adolescent mood clinic, USA **Participants:** *n* = 6 **Ages:** 14–17 **Gender:** 3 males (50%), 3 females (50%) **Diagnosis:** Depressive disorder based on K-SADS or CDRS-R (≥ 45)	Behavioural Activation **Duration:** 22 sessions over 18 weeks **Delivery:** Individual, face-to-face (2 doctoral level staff, 1 graduate student) ***n*** = 6 **Manual:** Yes	N/A	**Depression measures:** K-SADS CDRS-R BDI-II **Anxiety measures:** N/A **QoL measures:** N/A **Timings:** Pre-treatment Post-treatment
Ruggiero et al. (2005) [38]	**Setting:** Treatment Centre, USA **Participants*:**** n* = 1 **Ages:** 17 **Gender:** Female **Diagnosis:** Mild depression as measured using the BDI	Behavioral Activation Treatment for Depression (BATD) **Duration:** 8 sessions delivered weekly **Delivery:** Individual, face-to-face (Undergraduate student) ***n* = **1 **Manual:** Yes	N/A	**Depression measures:** BDI **Anxiety measures:** N/A **QoL measures:** N/A **Timings:** Pre-treatment Session 6 Session 8
Shadan et al. (2021) [50]	**Setting:** Outpatients Psychiatry Clinic, Dubai, UEA **Participants:** *n* = 1 **Ages:** 12 **Gender:** Female **Diagnosis:** MDD diagnosed by a psychiatrist and based on DSM-5	Behavioural Activation **Duration:** Initially delivered fortnightly and then monthly, number of sessions not reported **Delivery:** Individual, alternating face-to-face in person and virtual appointments (clinician type not specified) ***n* = **1 **Manual:** No	N/A	**Depression measures:** MFQ-P **Anxiety measures:** SCARED-C **QoL measures:** N/A **Timings:** Pre-treatment 4-weeks follow-up 6-weeks follow-up
Wallis et al. (2012) [51]	**Setting:** Local mental health service, Australia **Participants:** *n* = 5 **Ages:** 14–15 **Gender:** All female **Diagnosis:** Depressive disorder based on CES-D (no cut-offs specified)	Behavioural Activation **Duration:** 10 sessions over 10 weeks **Delivery:** Individuals, face-to-face (2 social workers) ***n*** = 5 **Manual:** Yes (as well as workbooks)	N/A	**Depression measures:** CES-D BDI-II **Anxiety measures:** N/A **QoL measures:** N/A **Timings:** BDI-II Only: Pre-treatment 2 weeks 3 weeks 6 weeks Completion (10 weeks)
Weersing et al. (2008) [52]	**Setting:** Primary care practice, USA **Participants:** *n* = 2 **Ages:** 13 and 17 **Gender:** 1 male (50%), 1 female (50%) **Diagnosis:** Depressive disorder based on CDI (≥ 13) or anxiety disorder SCARED (≥ 25)	Integrated brief behavioural therapy for anxiety and depression **Duration:** 8 30-min sessions over 12 weeks **Delivery:** Individual, face-to-face (mental health specialists) ***n*** = 2 **Manual:** Yes	N/A	**Depression measures:** CDI-P CDI-C K-SADS **Anxiety measures:** SCARED-P SCARED-C **QoL measures:** N/A **Timings:** Pre-treatment Post-treatment (12 weeks) 24-week follow-up

‘P’ denotes where a parent has completed a measure, ‘C’ denotes where a young person has completed a measure. MDD: Major Depressive Disorder. *Depression Measures:* CBCL: Child Behavior Checklist [55]; CDI-SF: Children’s Depression Inventory 2 [56]; CDRS-R: Children’s depression rating scale – revised [30]; CES-D: Center for epidemiologic studies depression scale [57]; CDI: Children’s depression Inventory [56]; CDS: Children’s Depression Scale [58]; BDI-II: Beck Depression Inventory [59]; K-SADS: Kiddie Schedule for affective disorders [54]; K-SADS-PL Kiddie Schedule for Affective Disorders and Schizophrenia Present and Lifetime Version [54]; MFQ: Mood and Feelings Questionnaire (MFQ) [60]; MINI-KID: Mini International Neuropsychiatric Interview for Children and Adolescents [61]; PHQ2: Patient Health Questionnaire-2 [62]; PROMIS depression scale: Patient Reported Outcomes Measurement Information System Depression Scale [63]; SMFQ: Short Mood and Feelings Questionnaire [64]; Anxiety and Depression Measures: RCADS: Revised Children’s and Depression Scale [65]; *Anxiety Measures:* ADIS-IV CSR: Anxiety disorders interview schedule for DSM-IV child interview Clinician Severity Rating [66]; GAD7: Generalized Anxiety Disorder-7 [67]; LSAS: Liebowitz Social Anxiety Scale [68]; MASC: Multi-dimensional anxiety scale for children [69]; SCARED: Screen for anxiety related emotional disorders [70]; RCMAS: Revised children’s manifest anxiety scale [71]; ADIS-IV: Anxiety disorders interview schedule for DSM-IV child interview [66]; *QoL Measures:* FQOLS: The Family Quality of Life Scale–Family Interactions Subscale [72]; KIDSCREEN-10 index [73]

### Quality assessments

#### Quality of RCTs

**Fig. 2:**
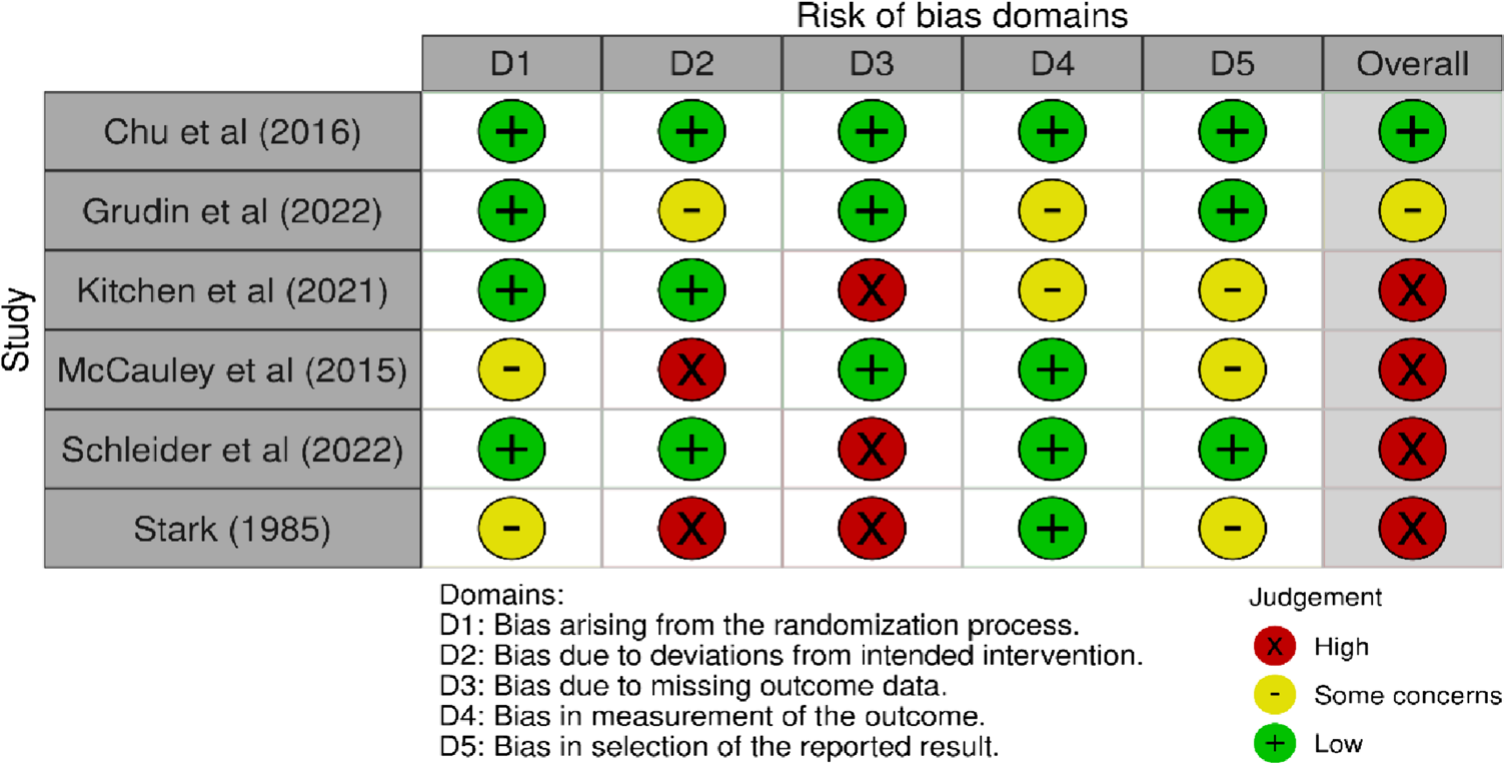
*RoB-2 included RCTs (n* = *6*

Using RoB-2, only Chu et al. [33] was found to have low risk of bias across all 5 assessed domains. There were ‘some concerns’ of bias in Grudin et al. [34], whereas the remaining four RCTs [16, 23, 32, 35] had a high risk of bias overall (Fig. 2). The domains with lowest scores for risk were ‘randomisation process’ and ‘bias in outcome measurements;’ the domain with the highest scores was ‘bias due to missing outcome data.’

The Moncrieff Scale scores for the RCTs (Table 2) ranged from 26 [16] to 38 [23]. All studies received two points for method of allocation, use of diagnostic criteria, recording exclusion criteria, describing outcome measures clearly, presenting results with inclusion of data for re-analysis of main outcomes and providing justified conclusions. All but one study [35] attained two points for providing objectives, specifications and main outcomes a priori and employing a representative sample. The domains with the highest risk of bias were for ‘conducting assessments of treatment compliance’ and ‘providing information on comparability and adjustment for difference in analysis.

**Table 2:** Moncrieff Scale – included RCTs (*n* = 6)

Domain	RCTs					
	Chu et al. (2016)	Grudin et al. (2022)	Kitchen et al. (2021)	McCauley et al. (2015)	Schleider et al. (2022)	Stark (1985)
Objectives and specifications, main outcomes a priori	2	2	2	1	2	2
Adequate sample size	2	0	0	2	2	0
Appropriate duration of trial and follow-up	1	1	2	2	1	1
Power calculations	0	2	0	2	2	0
Method of allocation	2	2	2	2	2	2
Concealment of allocation	0	2	2	2	2	0
Clear description of treatments	2	2	0	2	2	2
Blinding of subjects	N/A	N/A	N/A	N/A	N/A	N/A
Sources of subjects/representative sample	2	2	2	1	2	2
Use of diagnostic criteria	2	2	2	2	2	2
Record of exclusion criteria	2	2	2	2	2	2
Description of sample demographics	2	1	0	1	2	2
Blinding of assessor	2	2	0	1	2	1
Assessment of compliance with treatments	1	1	1	1	0	1
Details of side-effects	2	2	2	0	0	0
Record of number and reasons for withdrawal	1	2	1	2	1	2
Outcome measures described clearly	2	2	2	2	2	2
Information on comparability and adjustment for difference in analysis	1	0	0	2	0	2
Inclusion of all subjects in analysis (ITT)	2	2	0	2	2	2
Presentation of results with inclusion of data for re-analysis of main outcomes	2	2	2	2	2	2
Appropriate statistical analysis	1	2	0	1	2	1
Conclusions justified	2	2	2	2	2	2
Declarations of interest	2	2	2	2	2	0
Total	35	37	26	36	38	**30**

Maximum total score is 56; higher scores denote lower bias

#### Quality of pre-post evaluations

Moncrieff Scale scores for the pre-post evaluations ranged from 8 to 28 (Table 3). Only one domain—‘outcome measures described clearly’—attained two points across all studies. Most studies (15/18) attained two points for providing clear descriptions about treatments and sample demographics. None conducted power calculations, blinded assessors, or provided information about side-effects. Furthermore, the Moncrieff Scale guidelines stipulate that samples of < 50 participants receive a score of zero [27]. As the maximum sample size employed in the pre-post evaluations was 36 all received zero on this domain. Across the remaining domains, most studies attained a score of one or two suggesting measures had been taken to minimise bias.

**Table 3:** Moncrieff Scale – included pre-post evaluations (*n* = 18)

Domain	Pre-post evaluations						
	Arnott et al. (2020)	Brett et al. (2020)	Chu et al. (2009)	Douleh et al. (2013)	Dubicka et al. (2022)	Jacob et al. (2013)	Jenness et al. (2023)
Objections and specifications, main outcomes a priori	2	1	2	2	2	1	1
Adequate sample size	0	0	0	0	0	0	0
Appropriate duration of trial and follow-up	1	0	1	1	0	1	2
Power calculations	0	0	0	0	0	0	0
Method of allocation	1	N/A	N/A	N/A	N/A	N/A	N/A
Concealment of allocation	N/A	N/A	N/A	N/A	N/A	N/A	N/A
Clear description of treatments	2	2	2	2	2	2	2
Blinding of subjects	N/A	N/A	N/A	N/A	N/A	N/A	N/A
Sources of subjects/representative sample	2	1	1	1	1	1	1
Use of diagnostic criteria	2	1	1	2	1	2	0
Record of exclusion criteria	2	0	1	2	2	2	0
Description of sample demographics	0	2	2	2	2	2	2
Blinding of assessor	0	0	0	0	0	0	0
Assessment of compliance with treatments	1	1	2	1	2	2	2
Details of side-effects	0	0	0	0	0	0	0
Record of number and reasons for withdrawal	1	N/A	2	1	2	2	0
Outcome measures described clearly	2	2	2	2	2	2	2
Information on comparability and adjustment for difference in analysis	N/A	0	0	1	0	0	0
Inclusion of all subjects in analysis (ITT)	2	2	2	2	0	2	2
Presentation of results with inclusion of data for re-analysis of main outcomes	2	1	1	1	2	1	1
Appropriate statistical analysis	1	1	0	1	1	0	0
Conclusions justified	2	2	2	1	1	2	2
Declarations of interest	2	2	0	0	2	0	2
Total	**25**	**18**	**21**	**22**	**22**	**22**	**19**

Domain		Pre-post evaluations					
	Mohamed et al. (2024)	Nabors et al. (2021)	Pass, Hodgson, et al. (2018)	Pass et al. (2016)	Riley & Gaynor (2014)	Ritschel et al. (2016)	Ritschel et al. (2011)
Objections and specifications, main outcomes a priori	1	1	1	1	2	2	1
Adequate sample size	0	0	0	0	0	0	0
Appropriate duration of trial and follow-up	0	1	0	1	0	1	1
Power calculations	0	0	0	0	0	0	0
Method of allocation	N/A	N/A	N/A	N/A	N/A	N/A	N/A
Concealment of allocation	N/A	N/A	N/A	N/A	N/A	N/A	N/A
Clear description of treatments	2	2	2	2	2	2	2
Blinding of subjects	N/A	N/A	N/A	N/A	N/A	N/A	N/A
Sources of subjects/representative sample	1	1	1	1	1	1	1
Use of diagnostic criteria	2	1	2	2	2	2	2
Record of exclusion criteria	2	0	0	0	2	2	2
Description of sample demographics	1	2	2	2	2	2	2
Blinding of assessor	0	0	0	0	0	0	0
Assessment of compliance with treatments	2	2	2	2	2	2	2
Details of side-effects	0	0	0	0	0	0	0
Record of number and reasons for withdrawal	1	0	0	0	1	2	2
Outcome measures described clearly	2	2	2	2	2	2	2
Information on comparability and adjustment for difference in analysis	0	0	0	0	1	2	0
Inclusion of all subjects in analysis (ITT)	0	2	2	2	2	0	2
Presentation of results with inclusion of data for re-analysis of main outcomes	1	1	1	1	1	2	1
Appropriate statistical analysis	1	1	0	0	1	2	1
Conclusions justified	2	2	2	2	2	2	2
Declarations of interest	2	0	0	2	2	2	0
Total	20	18	17	20	**25**	**28**	**23**

Domain		Pre-post evaluations		
	Ruggiero et al. (2005)	Shadan et al. (2021)	Wallis et al. (2012)	Weersing et al. (2008)
Objections and specifications, main outcomes a priori	1	1	1	1
Adequate sample size	0	0	0	0
Appropriate duration of trial and follow-up	0	1	0	2
Power calculations	0	0	0	0
Method of allocation	N/A	N/A	N/A	N/A
Concealment of allocation	N/A	N/A	N/A	N/A
Clear description of treatments	2	1	0	2
Blinding of subjects	N/A	N/A	N/A	N/A
Sources of subjects/representative sample	1	1	1	1
Use of diagnostic criteria	2	1	1	1
Record of exclusion criteria	0	0	1	1
Description of sample demographics	2	2	0	2
Blinding of assessor	0	0	0	0
Assessment of compliance with treatments	1	1	0	0
Details of side-effects	0	0	0	0
Record of number and reasons for withdrawal	0	0	2	0
Outcome measures described clearly	2	2	2	2
Information on comparability and adjustment for difference in analysis	0	0	0	0
Inclusion of all subjects in analysis (ITT)	2	2	0	0
Presentation of results with inclusion of data for re-analysis of main outcomes	0	0	0	0
Appropriate statistical analysis	0	0	0	0
Conclusions justified	2	2	0	2
Declarations of interest	0	2	0	0
Total	15	14	8	14

Maximum total score is 56; higher scores denote lower bias

### Depression outcomes

Supplementary information S3 and S4 give details of individual study results. Below we summarise results across studies for each outcome measure.

#### Depression outcomes from RCTs

*CDRS-R*: Reductions in CDRS-R scores were demonstrated in four RCTs [32–35]. In Grudin et al. [34] scores reduced from pre-to-post-treatment, and then again at 3-months follow-up, in both the guided and self-guided BA groups. In the usual care group, although CDRS-R scores reduced from pre-to-post-treatment, little further reduction was seen at 3-months follow-up. Across time points these reductions were significant for the guided BA group (*B* =  − 11.3, *p* < 0.001, 95%CI − 14.9 to − 7.7) and the self-guided BA group (*B* =  *− *10.38, *p* < 0.001, 95%CI − 13.93 to − 6.82), but not usual care (*B* =  *− *4.40, *p* = 0.077, 95%CI − 9.33 to 0.52, *p* > 0.05).

In McCauley et al. [35] 76% of those randomised to BA scored ≤ 40 at post-treatment, indicating a depression diagnosis to be either ‘unlikely’ or ‘possible’, compared to 42% of the usual care group. Chu et al. [33] reported CDRS-R scores reduced from pre-to-post-treatment in the BA group, and increased in the wait-list group but statistical analyses were not performed. Finally, in Stark [32], CDRS-R scores reduced across time-points in all groups (BA, Self-Control Therapy, wait-list). Reductions were the greatest in the Self-Control Therapy group, followed by the BA group. The difference between groups at post-treatment was not significant (*p* < 0.30).

MFQ/SMFQ: Kitchen et al. [16] administered the Mood and Feelings Questionnaire (MFQ) which generates scores between 0–66 with higher scores representing worse mood. Larger mean reductions were reported from baseline to 6-months follow-up in the BA group (-18.11: *n* = 11, *M* = 33.91, *SD*: 11.80 to *n* = 5, *M* = 15.8, *SD*:6.22) compared to usual care (-8.8: *n* = 11, *M* = 35.55, *SD*:11.09 to *n* = 6, *M* = 26.67, *SD*: 12.6) on this measure. Grudin et al. [34] reported significant reductions on the Short Mood and Feelings Questionnaire (SMFQ) scores across all groups (guided-BA: *B* =  *− *4.4, *p* < 0.001, 95%CI − 6.2 to − 2.6; self-guided BA: *B* =  *− *3.39, *p* < 0.05, 95%CI − 6.48 to − 0.30; usual care: *B* =  *− *4.04, *p* = 0.001, 95%CI − 6.22 to − 1.86). In McCauley et al. [35] SMFQ scores reduced for both BA and usual care from pre-to-post-treatment, but with no statistical significance (*p* = 0.53).

*CDI:* In Stark [32] CDI scores reduced across all groups from pre-to-post-treatment and then to follow-up, with greater reductions seen in the BA and Self-Control group (ANCOVA test *p* < 0.01). Similar results were found by Schleider [23] who administered the short-form CDI (CDI-SF) and reported reductions across all groups from baseline to 3-months follow-up. Compared to the control group, those in the BA group and the active comparator group (Growth Mindset: GM) demonstrated significant decreases in depression from baseline to follow-up (BA: *t*(1,673) =  − 3.62; *P*_adj_ < 0.001; *d* = 0.18; 95% CI 0.08 to 028, GM: (*t*(1,629) =  − 3.53; *P*_adj_ < 0.001; *d* = 0.18; 95% CI, 0.08 to 0.27). No significant differences from baseline to follow-up were found between the two active treatment conditions.

**Fig. 3:**
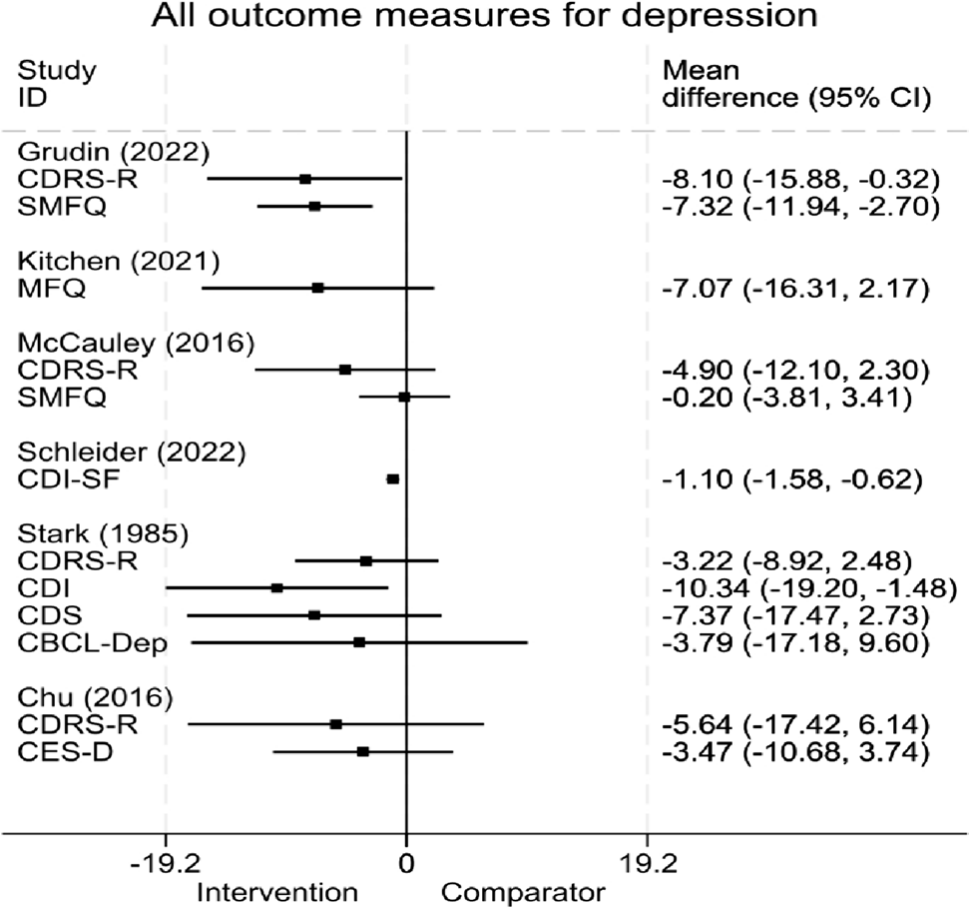
Forest plot of outcome measures for depression, by study

*CDS:* Stark [32] also reported reductions in CDS scores across all groups from pre-to-post-treatment and then to follow-up, with no statistical significance found between groups (*p* < 0.07). Chu et al. [33] reported larger reductions in CES-D scores for the BA group than the wait-list group but statistical tests were not performed. Figure 3 provides a graphical display of all depression outcome measures across RCTs.

**Fig. 4:**
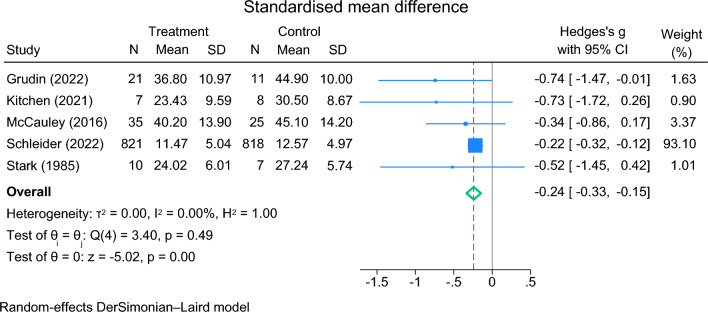
Forest plot of standardised mean difference (excluding Chu et al., 2016)

Except for Chu et al. [33], all RCTs reported the number of participants and mean (SD) of outcome measures by group. We therefore calculated the standardised mean differences (SMD) for continuous measures using Hedge’s adjusted g [74]. For the studies employing the CDRS-R, this measure was selected for estimating SMD. The remaining two studies [16, 23] employed only one depression measure each (MFQ, CDI-SF, respectively) which were used to calculate the SMD. The overall effect size was -0.24 (95% CI -0.33 to -0.15) suggesting a significant effect favouring the intervention (Fig. 4). According to Cohen’s *d* approach [75], an effect size of 0.5 could be considered moderate and 0.2 small. The I^2^ statistic was 0% (*p* = 0.49) indicating no statistical heterogeneity was present.

**Fig. 5:**
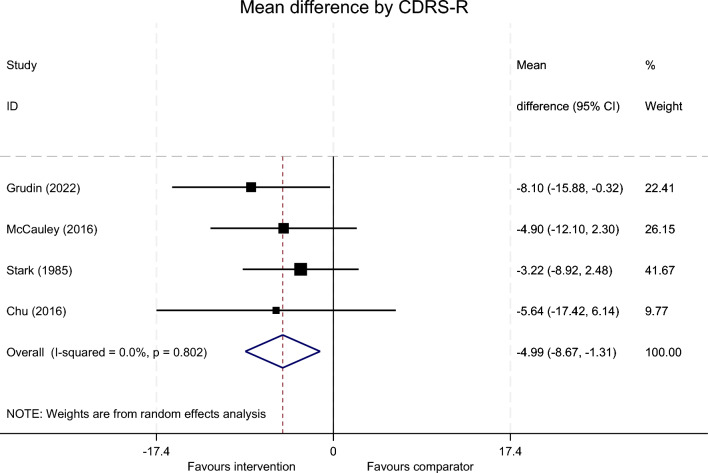
Random effects meta-analysis of the CDRS-R across included RCTs (n = 4)

Using the CDRS-R, a meta-analysis included the four studies [32–35] (Fig. 5). The overall mean difference was -4.99 (95%CI -8.67 to -1.31) in favour of the intervention (*Z* = 2.66, *p* = 0.008). Estimates of between-study variance τ^2^ = 0.0000. The I^2^ statistic was 0% (*p* = 0.802) suggesting no statistical heterogeneity.

Publication bias was examined using funnel plots. Given the limited number of studies, the conclusions are indicative but uncertain. There was no evidence of publication bias when using CDRS-R as the outcome measure (Supplementary Information S5) but there was some indication when examining the outcome measures using SMDs, (Supplementary Information S6).

#### Depression outcomes from pre-post evaluations

CDRS-R: Six of the pre-post evaluations [40, 42, 43, 45, 48, 49] administered the CDRS-R. In the two studies employing a stepped-care approach [40, 42] CDRS-R scores reduced for those receiving BA. Riley and Gaynor [42] found a clinically significant change on CDRS-R at the end of treatment as well as from post-non-directive therapy (NDT) to post-BA (*M* = 41.57, *SD:*11.79; *Z* = 2.37, *p* = 0.02). In Douleh [40] only one participant received BA and demonstrated reduced CDRS-R scores from pre-to-post-treatment and then follow-up where scores indicated a depression diagnosis to be ‘unlikely’ or ‘possible’. Although the participant in Nabors et al. [48] had reduced CDRS-R scores from pre-to-post-treatment, their post-treatment score still suggested diagnosable depression. Significant decreases in CDRS-R scores were seen from pre-to-post-treatment in the studies by Ritschel et al. [43] (*F*(2, 40) = 33.60, *p* < 0.001) and Ritschel et al. [49] (*F*(1,5) = 19.94, *p* < 0.01). The remaining pre-post evaluation to administer the CDRS-R [46], also reported reductions in scores from pre-to-post-treatment.

*BDI-II:* The studies which administered the BDI-II [38, 40, 46, 49, 51, 53] reported reductions following BA with statistical significance found in Ritschel et al. [47](*F*(1,5) = 330.00, *p* < *0.0*01) and Ritschel et al. (2016) (*F*(2,40) = 34.14, *p* < *0.0*01).

*RCADS depression subscale*: Statistically significant reductions from pre-treatment to follow-up we reported in two studies [17, 18].

*K-SADS:* Three studies [35, 46, 49] collected the K-SADS at pre-treatment and follow-up. McCauley et al. [35] found that 77% of BA participants no longer met diagnostic criteria for depression on the K-SADS post-treatment compared to 25% of those who received usual care. In Ritschel et al. [49] and Jacob et al. [46] four out of six participants and two out of three participants (respectively) no longer met criteria for MDD.

Across other depression measures completed by the pre-post evaluations, reductions in depression were reported from pre-treatment to follow-up. However, in Arnott et al., [39] while mean SMFQ scores reduced from pre-to-post-treatment, they increased at follow-up.

#### Parent-reported depression outcomes

Two RCTs [16, 34] and five pre-post evaluations [17, 18, 45, 52, 53] administered depression measures to parents/guardians which provided a proxy outcome for their children.

*MFQ/SMFQ*: In Kitchen et al. [16] the MFQ was administered to parents/guardians where a young person was ≤ 15 years. Reductions were reported from baseline to 6-months follow-up in both groups (BA group: -18.83; usual care: -2). These reductions were of a similar level to the child-reported outcomes. Grudin et al. [34] reported significant reductions in parent-reported SMFQ scores across all groups (guided-BA: *B* =  *− *2.83, *p* < 0.01, 95%CI − 4.31 to − 1.34, self-guided BA: *B* =  *− *3.75, *p* < 0.01, 95%CI − 5.65 to − 1.85; usual care: *B* =  *− *3.29,* p* < 0.01, 95%CI − 5.17 to − 1.42), mirroring the trend across the equivalent child-reported outcome scores*.*

*RCADS depression subscale*: Two studies [17, 18] demonstrated reduced scores between pre-treatment and follow-up and one [53] from pre-to-post treatment. In two studies [17, 45] reductions were larger overall than for the equivalent child-reported measures.

*CES-D*: In Chu et al. [45], the mean reduction in parent-reported CES-D score was almost twice as large (8.83) as for the child-reported score (4.55).

*CDI*: In Weersing et al. [52] parent-reported CDI scores for one participant, decreased from pre-to-post-treatment and increased at follow-up, whereas the child-reported score decreased at follow-up.

### Comorbid anxiety outcomes

Three RCTs [23, 32, 33] and five pre-post evaluations [45, 47, 50, 52, 53] assessed anxiety. Using the Screen for Anxiety and Related Emotional Disorders (SCARED), Chu et al. [33] reported greater reductions in scores from pre-to-post-treatment for the BA group than the waiting list control but with no statistical analyses. Furthermore, Jenness et al. [47] reported a reduction from pre-to-post-treatment, whilst both participants in Weersing et al. [52] had reductions in scores from pre-to-post-treatment and 6-months follow-up, with one attaining a score of zero at follow-up.

One RCT [33] and two pre-post evaluations [50, 52] also administered SCARED to parents/guardians. In Chu et al. [33] the results, as with the child-reported outcomes, showed greater reductions in anxiety scores in the BA group from pre-to-post-treatment compared to the waiting list group, with no statistical analyses performed. The mean pre-treatment score for the parent-reported SCARED (29.67) was nearly twice as high as the child-reported SCARED (16.89) with this gap persisting through to post-treatment. Shadan et al. [50] found reduced SCARED scores from baseline to week four and week six of treatment. Whilst Weersing et al. [52] reported reductions from pre-to-post-treatment, one participant attained an increased score at 6-months follow-up in contrast to the child-reported score which reduced to '0’.

Schleider et al. [23] reported reductions on the Generalized Anxiety Disorder-7 (GAD-7) across all groups from pre-treatment to 3-months follow-up. Compared to the control, the BA group did not show significant decreases in generalized anxiety (*t*(1,637) =  − 0.37; *p* = 0.72; *d* = 0.02; 95% CI, − 0.08, 0.12) from baseline to follow-up but the active comparator group (GM) did (*t(*1,629) =  − 2.08; *p* = 0.038; *d* = 0.10; 95% CI, 0.006, 0.20).

Stark [32] reported statistically significant reductions on Revised Children’s Manifest Anxiety Scale (RCMAS) scores from pre-to-post-treatment for those receiving BA or Self-Control Therapy (*p* < *0.0*1) and no improvement for the wait-list group. Individuals who received Self-Control Therapy demonstrated the highest reductions in anxiety at post-treatment.

Several other anxiety measures showed mixed results following BA. Jenness et al. [47] administered the Liebowitz Social Anxiety Scale (LSAS) and found that although there was a reduction in scores from pre-to-post-treatment, this remained in the clinical range. In Chu et al. [45] two of the five included participants demonstrated reduced anxiety scores following BA using the Multi-dimensional Anxiety Scale for Children (MASC). For the remaining three participants, one demonstrated increased anxiety, one remained the same whilst the other withdrew from treatment and did not complete follow-up. When administered to parents/guardians, Chu et al. [45] reported a mean reduction from pre-to-post-treatment which was significantly larger (12.58) than that for the child MASC (1.40).

Chu et al., [33] found the Anxiety Disorders Interview Schedule for DSM-IV child interview Clinician Severity Rating (ADIS-IV CRS) scores to be significantly lower in the BA group than the waiting list when anxiety was a secondary diagnosis (*B* = 2.09 (0.82), *p* = 0.01). Using the same measure, in Chu et al. [45] 75% of participants no longer met anxiety diagnosis criteria following BA.

Mohamed et al. [53], found scores on the RCADS anxiety subscale to reduce from pre-to-post treatment when parent-reported, however little change was seen in the child-reported scores.

### Combined depression and anxiety outcomes

One study [34] reported depression and anxiety scores combined using the RCADS. Scores reduced from pre-to-post-treatment in both the guided and self-guided BA groups, with increased scores then seen from post-treatment to follow-up. However, follow-up scores were lower in both groups than their respective values at pre-treatment. For the usual care group, RCADS scores increased from pre-to-post-treatment, reducing to around baseline levels at follow-up. These changes were significant in both BA groups (guided: *B* = -2.12, *p* < 0.001, 95%CI -0.92 to -3.32; self-guided: *B* = -2.48, *p* < 0.05, 95%CI –4.85 to -0.10) but not in the usual care group (*B* = -0.29, *p* = NS, 95%CI -2.75 to 2.16). On the parent-completed RCADS, scores reduced from pre-to-post-treatment and then, unlike the child-reported scores, continued to decrease through to follow-up across all groups. These improvements were significant in both BA groups (guided-BA group: *B* = -1.59, *p* < 0.05, 95%CI -2.94 to -0.24; self-guided BA group: *B* = -2.20, *p* < 0.01, 95%CI 3.58 to -0.82) but not the usual care group (*B* = 1.39, *p* = NS, 95%CI -3.17 to 0.39).

### Quality of Life (QoL) outcomes

Three studies [34, 40, 42] assessed QoL with two of these [34, 42] reporting their findings. Significant improvements in Health-Related QoL (HRQoL) were reported across both studies from baseline to end-of-treatment [42] and at 3-months follow-up [34] (see also Supplementary Information S7).

### Cost-effectiveness

We found no economic evaluations that met our inclusion criteria.

### Online and telephone-facilitated BA

Four included studies delivered BA remotely: two RCTs used exclusively an online format [23, 34] and two pre-post evaluations used videocall with some online materials [41, 53]. Three of the studies [34, 41, 53] assessed feasibility and one RCT assessed effectiveness [23] with a very large sample (*n* = 2,452). The collective findings support remotely delivered BA as an acceptable alternative to in-person BA (see also Supplementary Information S8).

The large RCT [23] found a small effect with a single online session. The authors concluded that the programme’s brevity and flexibility may have reached young people who might not have otherwise accessed help at all. The study was delivered in a self-selected group of young people from the general public, and it was not intended as a replacement for routine care in clinical populations.

## Discussion

This systematic review updated our earlier one [14] summarising study-level evidence on the effectiveness of BA for depression, comorbid anxiety and quality of life for children and young people. BA was delivered in-person, by phone or online, and was supported by a diverse group of trained staff in clinics, schools and the community. We looked for evidence on the cost-effectiveness of BA, but no economic evaluations met our inclusion criteria.

In a total of 24 studies, 11 more than our earlier review 7 years ago [14], the overall conclusion from 6 RCTs and 18 pre-post evaluations was that BA can reduce symptoms of depression in children and young people over time. The pre-post evaluations did not have a comparator, so we cannot say with certainty that this reduction was due to the intervention. In the RCTs, the reduction in depression symptoms was greater compared to waiting list controls, usual care or other therapeutic and supportive interventions. This was based on the CDRS-R, which was used in 4/6 RCTs and 6/18 pre-post evaluations.

Our meta-analysis of 4 RCTs found a small effect of BA compared to both active and passive controls. Only 1 of those RCTs was intended as a large clinical trial, whereas the other 3 were feasibility RCTs not designed or powered to test effectiveness. The large clinical trial was unusual in that it used a single session self-guided online BA during the pandemic as a public health intervention. This BA was not designed as a clinical intervention—despite the fact that the majority of the sample had elevated symptoms above a diagnostic cut-off; therefore, it is unlikely that this BA intervention is fit for the purpose of producing clinically meaningful change. Still, if we consider the small effect in the context of offering BA to the general population (e.g. all school children), then even a small difference per individual young person can shift the population distribution. In this case, studies need to be clear from the outset whether they intend to use BA as a clinical or a public health intervention.

In relation to whether BA for depression can also improve anxiety, the results were mixed and similar to findings reported in other studies [76]. Only a few studies measured and reported improvements on QoL. It is however challenging to detect any significant changes using QoL measures, moreover the guidelines for assessing QoL are less well-developed in children and adolescent populations than adult ones [77].

We identified three studies that used an online platform to facilitate BA delivery which suggested that these methods can help improve access to support for young people with depression. These findings endorse those of other studies that have found the remote delivery of BA to be acceptable and to enable the intervention to be delivered at large [78]. Besides increasing accessibility, delivering therapy to young people via an online platform has also been shown to increase anonymity [22] reduce stigmatisation and enable those reluctant to engage one-to-one with a therapist to access care [24].

Within the last 7 years, the number of young people participating in BA studies that meet our eligibility criteria, increased from 170 recruited from two countries – USA and Australia, to 2,758 participants in three additional countries—UK, Sweden and UAE. There have been studies in low middle income countries (LMICs) that evaluated BA for depression in children and adolescents [79, 80] but none met our inclusion criteria on account of their sample (not all participants had diagnosable depression) or their intervention (BA was only one of a multicomponent programme).

The depression outcomes reported within this review add support to consideration of BA as a promising treatment option for young people experiencing depression. Across the RCTs, improvements in depression were greater for those who received BA, or equivalent to groups who received an active comparator. These findings are similar to those reported in adult studies [81, 82].

Many of the included studies (*n* = 9/24) delivered BA in educational or community settings—rather than a clinic—supported by a wide range of professionals, including social workers, graduate students and school counsellors. This is important evidence to support policy initiatives that aim to expand evidence-based mental health interventions for young people beyond health services [4]. The findings are consistent with research in adult populations that found BA to be effective and cost-saving when delivered by non-specialists or by junior workers [6, 9].

One final significant observation was the discrepancies between parent and child reported outcomes in several of the included studies. These findings are consistent with other literature [83–85] in which parents either over-reported or under-reported depression symptoms by proxy for their children. This underscores the importance for researchers and clinicians to prioritise child self-reported outcomes for depression and anxiety, and not to rely exclusively or heavily on parental proxy report. Still, the parent’s view of their child’s depression symptoms is valuable as it can influence help-seeking in the first place and engaging in therapy thereafter.

### Limitations

The included 6 RCTs did not rate well on RoB-2, with only one [33] scoring 'low' risk of bias overall. Only 3/6 RCTs [23, 34, 35] conducted power calculations. Sample sizes were small in 5 RCTs (22–60 participants), whereas the sample of the sixth RCT was nearly 14 times larger than the combined sample of the rest. For the pre-post evaluations, no studies reported power calculations, blind assessment, or adverse effects. Across all 24 studies, only 5 had a follow-up period long enough for assessment of long-term outcomes as defined by the Moncrieff scale (i.e., ≥ 6 months); therefore, we cannot tell whether the effect of BA is durable.

We have included a narrative synthesis of findings relating to anxiety and mixed anxiety and depression, but these were considered as the “secondary effect” of BA, which is designed for depression. To answer the question of whether BA is effective for anxiety, a review needs to include studies in which participants were recruited based on diagnosable anxiety – with or without depression, or studies with mixed samples in which anxiety scores were reported at baseline and followed-up separately for a sub-sample of participants who scored above a clinical cut-off. In addition, we need a plausible theoretical or clinical framework that explains how BA may work for anxiety, given that its mechanism of action hinges on depression.

Finally, we were stringent in our inclusion criteria by selecting studies in which participants in the sample had diagnosable depression established by a validated screening tool or diagnostic manual. This meant however that we excluded many studies that used a mixed sample of participants from the general population, that included some young people with depression. This is because, these studies did not analyse and report the findings for those with depression separately e.g. Lynch et al. [86].

## Conclusion

BA is a promising enough intervention for reducing depression symptoms in children and adolescents to justify the need for further RCTs, providing that five conditions are met: studies are powered to detect a minimal clinically important difference; BA materials are fit-for-purpose to produce clinically meaningful change; follow-ups are longer than 6 months; primary outcomes are child-reported; intervention costs, resource use and adverse events are reported.

### Clinical relevance and implications of systematic review and meta-analysis

Our review is a comprehensive synthesis of all current available research on behavioural activation—a brief psychological intervention—for diagnosable depression in children and adolescents. It has two important implications. Firstly, it justifies the merit of future clinical trials and economic evaluations on behavioural activation for people younger than 18 years old. Secondly, it identifies five requirements for the design of future clinical trials to be able to generate evidence about the intervention’s clinical utility and value for money in this population.

## Conflicts of interest

None.

## Ethics approval

Not applicable.

## Informed consent

Not applicable.

## Consent for publication

Not applicable.

## Supplementary Information

Below is the link to the electronic supplementary material.

Supplementary file1 (DOCX 110 KB)

## Data Availability

Not applicable.
