# Supplementary material for: Is behavioural activation an effective treatment for depression in children and adolescents? An updated systematic review and meta-analysis
Source: Eur Child Adolesc Psychiatry. 2024 Apr 14;33(12):4133–56. doi: 10.1007/s00787-024-02429-3 (PMC11618157; doi:10.1007/s00787-024-02429-3)
Supplement: Supplementary file 1 — Supplementary file1 (DOCX 110 KB) [file 787_2024_2429_MOESM1_ESM.docx]

**S1. Search strategy**

<2015 to Present> 31/03/23

1 Behavio* activation.ti,ab.

2 (behavio* adj2 (intervention* or therap* or treatment* or psychotherap* or psycho-therap*)).ti,ab.

3 Behavior Therapy/

4 Cognitive Therapy/

5 self monitor*.ti,ab.

6 (Activit* adj3 (schedul* or plan* or arrang* or organis* or organiz*)).ti,ab.

7 1 or 2 or 3 or 4 or 5 or 6

8 Depression/

9 exp Depressive Disorder/

10 Depression.ti,ab.

11 Depressive.ti,ab.

12 Depressed.ti,ab.

13 ((low or negative or decreas*) adj2 (mood* or affect)).ti,ab.

14 8 or 9 or 10 or 11 or 12 or 13

15 7 and 14

16 young people.ti,ab.

17 young person*.ti,ab.

18 (child* or schoolchild*).ti,ab.

19 teen*.ti,ab.

20 adoles*.ti,ab.

21 youth*.ti,ab.

22 student*.ti,ab.

23 juvenile*.ti,ab.

24 pre-pubert*.ti,ab.

25 (pre-pubert* or prepubert*).ti,ab.

26 (pre-pubescen* or prepubescen*).ti,ab.

27 (pre-teen* or preteen*).ti,ab.

28 (puberty or pubertal).ti,ab.

29 Child/

30 Adolescent/

31 16 or 17 or 18 or 19 or 20 or 21 or 22 or 23 or 24 or 25 or 26 or 27 or 28 or 29 or 30

32 7 and 14 and 31

33 exp animals/ not humans.sh.

34 32 not 33

**S2. Reasons for exclusion**

**2015 review: Reasons for exclusion (n=32)**

| **Author(s) and year** | **Reason for exclusion** |
| --- | --- |
| Armento (2011) | <90% 18 years or under |
| Armento et al. (2012 | <90% 18 years or under |
| Bilek & Ehrenreich-May (2012) | Not a purely BA approach |
| Brent et al. (1999) | Not a purely BA approach |
| Brent et al. (1998) | Not a purely BA approach |
| Brent et al. (1997) | Not a purely BA approach |
| Chu et al. (2015) | Not a purely BA approach |
| Davidson et al. (2014) | Pre/post depression data not reported |
| Dundon (2010) | Not a behavioural treatment for depression |
| Esposito (2005) | Not a purely BA approach |
| Ettelson (2003) | Not a purely BA approach |
| Gawrysiak et al. (2009) | <90% 18 years or under |
| Harmon et al. (1980) | <90% 18 years or under |
| Kitchen et al. (2015) | Ongoing study |
| Kauer et al. (2011) | Not a purely BA approach |
| Landback et al. (2009) | Not a purely BA approach |
| Levin et al. (2010) | <90% 18 years or under |
| Ly et al. (2014) | <90% 18 years or under |
| Merry et al. (2004) | Not a purely BA approach |
| Mohammadi et al. (2013) | <90% 18 years or under |
| Moradveisi (2013) | <90% 18 years or under |
| Nystedt (1977) | Not a purely BA approach |
| Parker et al. (2011) | <90% 18 years or under |
| Pass et al. (2015) | Participants not diagnosed with depression at baseline |
| Proudfoot et al. (2013) | <90% 18 years or under |
| Reid et al. (2011) | Not a purely BA approach |
| Reynolds et al. (2011) | Participants not diagnosed with depression at baseline |
| Shaw et al. (1977) | <90% 18 years or under |
| Sobowale (2013) | Not a behavioural treatment for depression |
| Takagaki (2013) | <90% 18 years or under |
| Van Voorhees (2008) | Not a purely BA approach |
| Velayudhan (2010) | <90% 18 years or under |

**2023 review: Reasons for exclusion (n=48)**

| **Author(s) and year** | **Reason for exclusion** |
| --- | --- |
| Arjadi et al (2018) | ≤90% of population 18 years or under |
| Bamidele at al (2023) | Not a purely BA approach |
| Brent et al (2020) | Not all participants diagnosed with depression at baseline |
| Bryant et al (2022) | Not a purely BA approach |
| Chen et al (2021) | ≤90% of population 18 years or under |
| Chu et al (2015) | Not a behavioural treatment for depression |
| Collado et al (2016) | ≤90% of population 18 years or under |
| Crits-Christoph et al (2021) | ≤90% of population 18 years or under |
| Dingzhou et al (2018) | Full text unavailable |
| Fernandez-Rodriguez et al (2023) | ≤90% of population 18 years or under |
| Furukawa et al (2018) | ≤90% of population 18 years or under |
| Hemanny et al (2020) | ≤90% of population 18 years or under |
| Hemanny et al (2018) | ≤90% of population 18 years or under |
| Jelinek et al (2020) | ≤90% of population 18 years or under |
| Kanter et al (2015) | ≤90% of population 18 years or under |
| Lee et al (2022) | ≤90% of population 18 years or under |
| Li et al (2023) | ≤90% of population 18 years or under |
| Ly et al (2015) | ≤90% of population 18 years or under |
| Lynch et al (2021) | Not all participants diagnosed with depression at baseline |
| Martinez-Vispo et al (2020) | ≤90% of population 18 years or under |
| McCluskey (2018) | ≤90% of population 18 years or under |
| McIndoo et al (2016) | ≤90% of population 18 years or under |
| Meinzer et al (2021) | ≤90% of population 18 years or under |
| Mori et al (2016) | Not all participants diagnosed with depression at baseline |
| Oddo et al (2021) | ≤90% of population 18 years or under |
| Parra et al (2019) | ≤90% of population 18 years or under |
| Pass et al (2015) | Participant not diagnosed with depression at baseline |
| Pass, Sancho et al (2018) | Pre/post depression data not reported |
| Pass, Lejuez & Reynolds (2018) | Inclusion criteria not specified |
| Pott et al (2022) | ≤90% of population 18 years or under |
| Rasing et al (2018) | Not a purely BA approach |
| Schwartz (2018) | ≤90% of population 18 years or under |
| Shahar & Govrin (2017) | ≤90% of population 18 years or under |
| Shiota et al (2017) | Not all participants diagnosed with depression at baseline |
| Soleimani (2017) | ≤90% of population 18 years or under |
| Soucy et al (2017) | ≤90% of population 18 years or under |
| Stephens (2022) | Not a purely BA approach |
| Sugiyama et al (2019) | Not all participants diagnosed with depression at baseline |
| Suh et al (2021) | Not all participants diagnosed with depression at baseline |
| Szuhany & Otto (2020) | ≤90% of population 18 years or under |
| Takagaki et al (2021) | Not all participants diagnosed with depression at baseline |
| Takagaki et al (2018^a^) | ≤90% of population 18 years or under |
| Takagaki et al (2018^b^) | ≤90% of population 18 years or under |
| Takagaki et al (2016^a^) | Not all participants diagnosed with depression at baseline |
| Takagaki et al (2016^b^) | Not all participants diagnosed with depression at baseline |
| Tornivuori et al (2023) | Not a purely BA approach |
| Webb et al (2023) | Not all participants diagnosed with depression at baseline |
| Weersing (2017) | Not all participants diagnosed with depression at baseline |

**S3.** Results reported from the RCTs (n=6)

| **Study**  **Author/Year** | **Measure** | **Intervention Group** | | | **Comparator Group** | | | **Difference between groups** | **Change from pre-treatment within group** |
| --- | --- | --- | --- | --- | --- | --- | --- | --- | --- |
|  |  | **Pre-treatment (M (SD), n)** | **Post-treatment (M (SD), n)** | **Follow-up* (M, SD, n)** | **Pre-treatment (M (SD), n)** | **Post-treatment* (M (SD), n)** | **Follow-up* (M (SD) n)** |  |  |
| Chu et al (2016) [33] | CDRS-R | 42.57 (5.08), n=21 | 37.67 (5.36), n=21 | *NR* | 46.0 (3.95), n=14 | 57.0 (0.00), n=14 | *NA (received delayed treatment)* | Change from pre-to-post: N=13, adjusted mean difference -5.64 (SE 6.01), *p=*0.37 | *NR* |
|  | CES-D-P | 10.22 (2.08), n=21 | 9.58 (1.28), n=21 | *NR* | 12.91 (1.95), n=14 | 10.00 (1.85), n=14 | *NA (received delayed treatment)* | Change from pre-to-post: N=35, adjusted mean difference 2.27 (SE 2.59), p=0.38 | **Intervention, to follow-up:** n=21,  -0.74 (SE 1.14), p=0.52 |
|  | CES-D-C | 21.00 (2.15), n=21 | 16.38 (2.30), n=21 | *NR* | 20.22 (2.73), n=14 | 19.07 (3.15), n=14 | *NA (received delayed treatment)* | Change from pre-to-post: N=35, adjusted mean difference -3.47 (SE 3.68), p=0.35 | **Intervention, to follow-up:** n=21,  -2.23 (SE1.18), p=0.07 |
|  | ADIS-IV^A^  ADIS principal diagnosis CSR | 4.62 (0.27), n=21 | 2.48 (0.40), n=21 | *NR* | 4.93 (0.31), n=14 | 3.79 (0.61), n=14 | *NA (received delayed treatment)* | Change from pre-to-post: N=35, adjusted mean difference -1.00 (SE 0.71), p=0.16 | **Intervention, to follow-up:** n=21,  -1.32 (0.21), p=0.000 |
|  | ADIS secondary diagnosis CSR | 3.82 (0.32), n=21 | 1.53 (0.55), n=21 | *NR* | 4.6 (0.34), n=14 | 4.4 (0.54), n=14 | *NA (received delayed treatment)* | Change from pre-to-post: N=27, adjusted mean difference -2.09 (SE 0.82), *p*=0.01 | **Intervention, to follow-up:** n=17,  -1.58 (0.23), p=0.000 |
|  | SCARED-P^A^ | 16.89 (2.14), n=21 | 12.09 (1.95), n=21 | *NR* | 18.64 (2.59), n=14 | 17.43 (2.89), n=14 | *NA (received delayed treatment)* | Change from pre-to-post: N=35, adjusted mean difference -3.69 (SE 3.81), p=0.34 | **Intervention, to follow-up:** n=21,  -3.85 (1.16), p=0.004 |
|  | SCARED-C^A^ | 29.67 (2.23), n=21 | 21.05 (2.41), n=21 | *NR* | 28.51 (3.36), n=14 | 26.93 (4.56), n=14 | *NA (received delayed treatment)* | Change from pre-to-post: N=35, adjusted mean difference -7.03 (SE 5.24), p=0.18 | **Intervention, to follow-up:** n=21,  -4.75 (1.68), p=0.01 |
| Grudin et al (2022) [34] | CDRS-R | **Group 1:** Therapist guided BA 52.2 (9.4), n=11  **Group 2:** self-guided BA 55.1 (10.2), n=10 | **Group 1:** Therapist guided BA 34.8 (10.1), n=11  **Group 2:** self-guided BA 39.0 (12.0), n=10 | **Group 1:** Therapist guided BA 29.1 (10.1), n=11  **Group 2:** self-guided BA 31.6 (11.0), n=10 | 53.2 (9.0), n=11 | 44.9 (10.0), n=11 | 44.6 (13.6), n=11 | *NR* | **To follow-up: Group 1:** -11.3 (95% CI -14.9 to -7.7), p<0.001  Group 2: -10.38 (95% CI -13.93 to -6.82), p<0.001  **Comparator:** -4.40 (95% CI -9.33 to 0.52), p>0.05 |
|  | SMFQ-P | **Group 1:** Therapist guided BA 11.6 (5.8), n=11  **Group 2:** self-guided BA 12.5 (5.9), n=10 | **Group 1:** Therapist guided BA 7.6 (5.0), n=11  **Group 2:** self-guided BA 5.5 (4.7), n=10 | **Group 1:** Therapist guided BA 5.7 (4.3), n=11  **Group 2:** self-guided BA 5.0 (3.6), n=10 | 15.2 (4.1), n=11 | 11.2 (7.2), n=11 | 9.3 (5.0), n=11 | *NR* | **To follow-up:**  **Group 1:** -2.83 (95% CI -4.31 to -1.34), p<0.01  **Group 2:** -3.75 (95% CI -5.65 to -1.85), p<0.01  **Comparator:** -3.29 (-5.17 to -1.42), p<0.01 |
|  | SMFQ-C | **Group 1:** Therapist guided BA 13.6 (5.4), n=11  **Group 2:** self-guided BA 13.9 (6.5), n=10 | **Group 1:** Therapist guided BA 6.2 (5.6), n=11  **Group 2:** self-guided BA 4.9 (5.5), n=10 | **Group 1:** Therapist guided BA 4.6 (4.5), n=11  **Group 2:** self-guided BA 8.3 (6.0), n=10 | 16.8 (6.2), n=11) | 12.9 (7.8), n=11 | 9.6 (5.8), n=11 | *NR* | **To follow-up:**  **Group 1:** -4.4 (95% CI -6.2 to -2.6), p<0.001  **Group 2:** -3.39, (95% CI -6.48 to -0.30), p<0.05  **Comparator:** -4.04 (95% CI -6.22 to -1.86), p=0.001 |
|  | RCADS-P | **Group 1:** Therapist guided BA 10.6 (6.6), n=11  **Group 2:** self-guided BA 10.3 (4.9), n=10 | **Group 1:** Therapist guided BA 9.2 (6.4), n=11  **Group 2:** self-guided BA 8.8 (4.1), n=10 | **Group 1:** Therapist guided BA 7.7 (6.1), N=11  **Group 2:** self-guided BA 6.5 (4.5), n=10 | 10.4 (7.0), n=11 | 10.0 (5.3), n=11 | 7.9 (5.7), n=11 | *NR* | **To follow-up:**  **Group 1:** -1.59 (95% CI -2.94 to -0.24)  **Group 2:** -2.20 (95% CI -3.58 to -0.82)  **Comparator:** -2.63 (95% CI -5.31 to 0.04) |
|  | RCADS-C | **Group 1:** Therapist guided BA 12.5 (4.3), n=11  **Group 2:** self-guided BA 14.4 (8.8), n=10 | **Group 1:** Therapist guided BA 7.9 (5.2), n=11  **Group 2:** self-guided BA 7.4 (4.6), n=10 | **Group 1:** Therapist guided BA 8.3 (5.3), n=11  **Group 2:** self-guided BA 10.1 (5.7) n=10 | 12.7 (6.2), n=11 | 15.5 (10.3), n=11 | 12.3 (9.0), n=11 | *NR* | **To follow-up:**  **Group 1:** -2.12 (95% CI -0.92 to -3.32)  **Group 2:** -2.48 (95% CI -4.85 to -0.10)  **Comparator:** -0.29 (95% CI -2.75 to 2.16) |
|  | KIDSCREEN-10-C | **Group 1:** Therapist guided BA 31.3 (5.3), n=11  **Group 2:** self-guided BA 30.7 (4.2), n=10 | **Group 1:** Therapist guided BA 34.4 (5.3), n=11  **Group 2:** self-guided BA 36.0 (6.2), n=10 | **Group 1:** Therapist guided BA 38.0 (5.1), n=11  **Group 2:** self-guided BA 38.3 (6.0), n=10 | 28.4 (4.2), n=11 | 30.4 (5.2), n=11 | 33.7 (4.9), n=11 | *NR* | **To follow-up:**  **Group 1:** 3.03 (95% CI 1.80 to 4.27)  **Group 2:** 3.46 (95% CI 1.93 to 4.99)  **Comparator:** 2.71 (95% CI 0.65 to 4.76) |
|  | KIDSCREEN-10-P | **Group 1:** Therapist guided BA 29.6 (4.2), n=11  **Group 2:** self-guided BA 30.3 (5.6), n=10 | **Group 1:** Therapist guided BA 31.5 (3.3), n=11  **Group 2:** self-guided BA 34.8 (4.4), n=10 | **Group 1:** Therapist guided BA 34.2 (3.4), n=11  **Group 2:** self-guided BA 33.0 (8.3), n=10 | 28.0 (5.4), n=11 | 31.2 (4.7), n=11 | 31.6 (5.0), n=11 | *NR* | **To follow-up:**  **Group 1:** 2.16 (95% CI 1.14 to 3.17)  **Group 2:** 2.25 (95% CI 0.74 to 3.76)  C**omparator:** 1.82 (95% CI 0.01 to 3.62) |
| Kitchen et al (2021) [16] | MFQ-C | 33.91 (11.8), n=11 | 23.43 (9.59), n=7 | 15.8 (6.22), n=5 | 35.55 (11.09), n=11 | 30.5 (8.67), n=8 | 26.67 (12.6), n=6 | *NR* | **To follow-up:**  **Intervention:** -18.11  **Comparator:** -8.8 |
|  | MFQ-P | 29.83 (7.36), n=6 | 29.33 (8.62), n=*NR* | 11 (1.41), n=*NR* | 29 (8.58), n=7 | 26.6 (15.79), n=*NR* | 27 (4.76), n=*NR* | *NR* | **To follow-up:**  **Intervention:**  -18.83  **Comparator:** -2 |
| McCauley et al (2015) [35] | CDRS-R | 57.6 (11.8), n=35 | 40.18 (13.91), n=35 | - | 57.84 (8.26), n=25 | 45.05 (14.23), n=25 | - | Change from pre-to-post: *p*=0.12 | **To post-treatment:**  **Intervention:**  -18.6 (95% CI -23.2, -13.9)  **Comparator:**  -13.1(95% CI -18.3, -7.8) |
|  | SMFQ | 16.05 (6.05), n=35 | 6.3 (7.4), n=35 | - | 15.64 (6.2), n=25 | 6.5 (6.5), n=25 | - | Change from pre-to-post: *p*=0.53 | **To post-treatment:**  **Intervention:** -9.6 (95% CI -12.3, -6.9)  **Comparator:** -9.2 (95% CI -12.7,-5.8) |
|  | MASC^A^ | *NR* | *NR* | *NR* | *NR* | *NR* | *NR* | *NR* | *NR* |
| Schleider et al (2022) [23] | CDI-SF | 14.15 (4.06), n=821 | - | 11.47 (5.04), n=821 | **Group 1:** GM SSI: 14.22 (4.13), n= 813  **Group 2:** Placebo control: 14.31 (4.12), n=818 | - | **Group 1:** GM SSI: 11.58 (5.08), n=813  **Group 2:** Placebo control: 12.57 (4.97), n=818 | From pre to follow-up:  Intervention vs Group 2: *t*(1,673)=−3.62; *p*<0.001; *d*=0.18; 95% CI 0.08, 0.28  Group 1 vs Group 2: *t*(1,629)=−3.53;*p*<0.001; *d*=0.18; 95% CI 0.08, 0.27  Intervention vs Group 1: *t*(1,632)=−0.20; *p* =0.845; *d*=-0.01; 95% CI −0.11, 0.09 | **To follow-up:**  **Intervention**: t(820)=-9.62, p<0.001; d=-0.47; 95% CI -0.54, -0.39  **Group 1:** t(812)=-12.29, p<0.001; d=-0.43, 95% CI -0.50, -0.36  **Group 2:** t(817)=-12.29, p<0.001; d=-0.34, 95% CI -0.41, -0.27 |
|  | GAD7^A^ | 2.96 (0.76), n=821 |  | 2.73 (0.82), n=821 | **Group 1:** GM SSI: 3.00 (0.74), n=813  **Group 2:** Placebo control: 3.01 (0.73), n=818 |  | **Group 1:** GM SSI: 2.68 (0.81), n=813  **Group 2:** Placebo control: 2.78 (0.79), n=818 | From pre to follow-up:  Intervention vs Group 2: *t*(1,637)=−0.37; *P*=0.72; *d*=0.02; 95% CI, −0.08, 0.12  Group 1 vs Group 2: *t(*1,629)=−2.08; *p*=0.038; *d*=0.10; 95% CI, 0.006, 0.20  Group 1 vs Intervention: t(1,632)=2.01, p=0.044; d=-0.10, 95% CI 0.002, 0.20 | *NR* |
| Stark (1985) [32] | CDRS-R | 33.50 (10.27), n=10 | 24.02 (6.01), n=10 | 24.28 (4.68), n=9 | **Group 1:** SCT  37.22 (8.36), n=9  **Group 2:** WL  27.57(3.51), n=9 | **Group 1:** SCT  22.90 (4.36), n=9  **Group 2:** WL  27.24(5.74), n=7 | **Group 1:** SCT  20.69 (3.45), n=8  Group 2: WL  22.60 (5.03), n=5 | Post-treatment: F=1.26, ns  Follow-up: F=6.36, p=0.02 | **Intervention:** Pre-to-post, n=10, d=9.40 (SD 9.32), t=3.19, p<0.05; pre to follow-up, n=9, d=9.44 (SD 8.10), t=3.50, p<0.01; post to follow-up, n=9, d=-0.33 (SD 3.74), t=0.27, ns  **Group 1:** Pre-to-post, n=9, d=13.22 (SD 7.89), t=5.03, p<0.001; pre to follow-up, n=8, d=15.38 (SD 6.46), t=6.73, p<0.001; post to follow-up, n=8, d=3.00 (SD 3.55), t=2.39, p<0.05  **Group 2:** Pre-to-post, n=7, d=1.86 (SD 4.38), t=1.12, ns; pre to follow-up, n=5, d=5.60 (SD 5.73), t=2.19, ns; post to follow-up, n=5, d=3.80 (SD 6.14), t=1.38, ns |
|  | CDI | 22.40 (8.47), n=10 | 9.11 (8.32), n=10 | 7.43 (7.23), n=9 | **Group 1:** SCT  21.60 (5.48), n=9  **Group 2:** WL  20.00 (10.71), n=9 | **Group 1:** SCT  8.09 (6.65), n=9  **Group 2:** WL  19.45 (10.31), n=7 | **Group 1:** SCT  5.36 (5.04), n=8  **Group 2:** WL  7.40 (5.68), n=5 | Post-treatment: F=6.01, p<0.01  Follow-up: F=0.48, ns | **Intervention:** Pre-to-post, n=10, d=15.10 (SD 12.45), t=4.18, p<0.01; pre to follow-up, n=9, d=20.44 (SD 13.67), t=5.58, p<0.001; post to follow-up, n=9, d=5.44 (SD 6.56), t=1.60, ns  **Group 1:** Pre-to-post, n=9, d=13.56 (SD 7.76), t=5.24, p<0.001; pre to follow-up, n=8, d=15.63 (SD 6.50), t=6.80, p<0.001; post to follow-up, n=8, d=4.13 (SD 3.48), t=3.35, p<0.01  **Group 2**: Pre-to-post, n=7, d=1.43 (SD 3.21), t=1.18, ns; pre to follow-up, n=5, d=8.40 (SD 7.09), t=2.65, p<0.06; post to follow-up, n=5, d=7.60 (SD 5.94), t=2.86, p<0.05 |
|  | CDS | 71.10 (10.38), n=10 | 55.24 (12.18), n=10 | 50.03 (13.23), n=9 | **Group 1:** SCT  72.40 (10.31), n=9  **Group 2:** WL  66.00 (18.80), n=9 | **Group 1:** SCT  50.29 (8.63), n=9  **Group 2:** WL  62.61 (7.14), n=7 | **Group 1:** SCT  46.46 (8.31), n=8  **Group 2:** WL  48.20 (13.29), n=5 | Post-treatment: F=2.99, ns  Follow-up: F=0.42, ns | **Intervention:** Pre-to-post, n=10, d=15.10 (SD 12.45), t=3.84, p<0.01; pre to follow-up, n=9, d=20.44 (SD 13.67), t=4.49, p<0.01; post to follow-up, n=9, d=5.44 (SD 6.56), t=2.49, p<0.05  **Group 1:** Pre-to-post, n=9, d=19.89 (SD 10.94), t=5.46, p<0.001; pre to follow-up, n=8, d=24.25 (SD 13.38), t=5.13, p<0.001; post to follow-up, n=8, d=5.88 (SD 9.33), t=1.78, ns  **Group 2**: Pre-to-post, n=7, d=6.29 (SD 4.07), t=4.09, p<0.01; pre to follow-up, n=5, d=10.80 (SD 15.17), t=1.59, ns; post to follow-up, n=5, d=4.20 (SD 11.26), t=0.83, ns |
|  | RCMAS^A^ | 18.30(3.43), n=10 | 15.37(5.75), n=10 | 6.77(6.14), n=9 | **Group 1**: SCT  17.40(3.81), n=9  **Group 2:** WL  14.71(5.47), n=7 | **Group 1:** SCT  8.80(3.68), n=9  **Group 2:** WL  15.02(7.37), n=7 | **Group 1:** SCT  6.75(8.08), n=8  **Group 2:** WL  5.60 (6.6), n=5 | Post-treatment: F=1.28, ns  Follow-up: F=0.00, ns | **Intervention:** Pre-to-post, n=10, d=5.30 (SD 4.64), t=3.61, p<0.01; pre to follow-up, n=10, d=11.67 (SD 6.76), t=5.17, p<0.001; post to follow-up, n=9, d=6.89 (SD 3.98), t=5.19, p<0.001  **Group 1:** Pre-to-post, n=9, d=8.89 (SD 4.70), t=5.67, p<0.001; pre to follow-up, n=9, d=11.88 (SD 8.87), t=3.79, p<0.01; post to follow-up, n=8, d=2.75 (SD 8.99), t=0.87, ns  **Group 2:** Pre-to-post, n=7, d=4.43 (SD 6.50), t=1.80, ns; pre to follow-up, n=7, d=6.80 (SD 4.55), t=3.34, p<0.05; post to follow-up, n=5, d=4.80 (SD 3.70), t=2.90, p<0.05 |

**Notes.** *Final follow-up completed. *NR*=Not reported. ^A^Anxiety measures, ^Q^QoL measures. *UC:* Usual Care; ^⤉^Scores not presented as numerical values, presented in a line graph and interpreted by review authors.

**S4.** Results reported in pre-post evaluations

| **Study Author/Year** | **Measure** | **Pre-treatment (M (SD), n)** | **Post-treatment (M (SD) n)** | **Follow-up***  **(M (SD), n)** | **Significance** |
| --- | --- | --- | --- | --- | --- |
| Arnott et al. (2020) [39] | SMFQ | 12 (9.05), n=4 | 5.8 (8.26), n=4 | 10.3 (11.37), n=3 |  |
| Brett et al. (2020)[44] | RCADS depression subscale | P1: 16  P2: 19 | P1: 5  P2: 7 | *NR* | P1: Reliable change criterion >6 change  P2: Reliable change criterion: >6 change |
| Chu et al. (2009) [45] | CES-D-P | P1:17  P2: 21  P3: -  P4: 31  P5:29  *M*= 24.5(6.61), n=4 | P1:15  P2:17  P3: -  P4: 15  P5: -  *M*= 15.67(1.16), n=3 | - | Mean reduction in CES-D-P scores of 8.83 from pre-to-post-treatment. |
|  | CES-D-C | P1: 27  P2: 38  P3: 42  P4: 35  P5: 42  *M*= 36.8(6.22), n=5 | P1:15  P2: 26  P3: -  P4: 46  P5: 42  *M*= 32.25 (14.39) | - | Mean reduction in CES-D-C scores of 4.55 from pre-to-post-treatment. |
|  | ADIS-IV CSR^A^ | 6.00(.71), n=5 | 3.25 (1.26), n=4 | - | Reductions from pre-to-post-treatment in ADIS CSR scores with 75% participants (¾) no longer meeting criteria for principal diagnosis at post-treatment |
|  | MASC-P^A^ | P1:38  P2: 55  P3: -  P4: 70  P5: 42  *M*=51.25(14.45), n=4 | P1:33  P2: 43  P3: -  P4: 40  P5: -  *M*= 38.67(5.13), n=3 | - | Mean reduction in MASC-P scores of 12.58 from pre-to-post-treatment. |
|  | MASC-C^A^ | P1:35  P2:61  P3:41  P4:67  P5:53  *M*=51.40(13.37), n=5 | P1:23  P2: 56  P3: -  P4: 68  P5: 53  *M*= 50(19.13), n=4 | - | Mean reduction in MASC-C scores of 1.40 from pre-to-post-treatment. |
| Douleh (2013) [40] | CDRS-R | 58.79 (9.11), n = 14 | 31 n = 1 | 25 n = 1 | Clinically significant improvement in depression on CDRS-R and BDI-II observed post-BA. |
|  | BDI-II | 21(11.48), n = 14 | 14 n = 1 | 11 n = 1 |  |
| Dubicka et al (2022) [41] | MFQ | 43.2 (9.3), n=36 | 27.6 (14.7), n=28 | - | n=16 showed a ≥10 points improvement on the MFQ; n=4, showed a ≥5 points improvement on the MFQ; n=1 showed a 3-point improvement on the MFQ, n=4 showed worsening of MFQ scores, these were not in line with clinical assessment or the young person’s narrative. |
| Jacob et al (2013) [46] | CDRS-R | P1:45  P2:72  P3:61  *M*=59.3(13.6) | P1:21  P2: 27  P3: 17  *M*=21.7(4.1) | - | 2/3 participants no longer met criteria for MDD at post-treatment |
|  | BDI-II | P1:21  P2:27  P3:17  *M*=21.7(4.1) | P1:4  P2:6  P3:2  *M*=4.0(2.0) | - |  |
| Jenness et al (2022) [47] | SMFQ | 15, n=1 | 5, n=1 | *NR* | By termination of treatment participant’s depression and generalised anxiety in remission. Depression remission sustained at 1-year follow-up |
|  | SCARED | 42, n=1 | 14, n=1 | *NR* |  |
|  | LSAS^A^ | 74, n=1 | 41, n=1 | *NR* |  |
| Mohamed et al (2024) [53] | BDI-II | 24.66, n=1 | 21.83, n=6 | *NR* | Post-intervention, 1/6 demonstrated a reliable change in depression scores. For anxiety all parent scores reduced for anxiety with 4/6 demonstrating a reliable change score (based on parent-report). Overall, 4/6 participants showed a reliable change score (i.e. reduction) for at least one clinical outcome (based on parent or self-report). |
|  | RCADS Depression subscale-P** | 18.33, n=6 | 17.66, n=6 | *NR* |  |
|  | RCADS Depression subscale-C** | 13.83, n=6 | 12.00, n=6 | *NR* |  |
|  | RCADS Anxiety subscales-P^A^** | 61.83, n=6 | 48.66, n=6 | *NR* |  |
|  | RCADS Anxiety subscales-C^A^** | 41.83, n=6 | 41.50, n=6 | *NR* |  |
| Nabors et al (2021) [48] | CDRS-R | 65, n=1 | 48, n=1 | - | Following BA the participant’s healthy behavior increased, depressed behavior decreased, and mood improved |
|  | PROMIS Depression scale | 64, n=1 | 50, n=1 | - |  |
| Pass, Hodgson et al (2018) [17] | RCADS Depression subscale- P | 110^⤉^,n=1 | 80^⤉^,n=1 | 60^⤉^,n=1 | Reliable and clinically significant improvement in RCADS scores from assessment to follow-up. |
|  | RCADS Depression subscale – C | 75^⤉^ ,n=1 | 68^⤉^,n=1 | 50^⤉^,n=1 |  |
|  | RCADS – P | *NR* | *NR* | *NR* | *NR* |
|  | RCADS – C | *NR* | *NR* | *NR* |  |
| Pass et al. (2016) [18] | RCADS Depression subscale- P | 18^⤉^ ,n=1 | 10^⤉^ ,n=1 | 6^⤉^ ,n=1 | Reliable and clinically significant improvement in RCADS Depression subscale scores from assessment to follow-up. |
|  | RCADS Depression subscale – C | 19^⤉^ ,n=1 | 12^⤉^ ,n=1 | 11^⤉^ ,n=1 |  |
| Riley and Gaynor (2014) [42] | CDRS-R | 55.36 (12.36), n = 11 | 41.57 (11.79), n = 7 | - | No significant change in depression symptoms from pre-treatment (CDRS-R: *M*=59.57, *SD*:13.29; CDI:*M=*25.57*, SD:*10.21) to post-NDT (CDRS-R *M=*58.86, *SD:*17.08, *Z=* −0.09, *p=*.93; CDI *M=* 24.71, *SD:*8.4, *Z=*−0.51, *p=*.61) in those who received BA (n=7). However, significant difference reported from post-NDT to post-BT (CDRS-R *M=* 41.57, *SD:*11.79, *Z=*−2.37, *p=*.02; CDI: *M=*16.29, *SD:*10.24, *Z=*−2.37, *p=*.02). |
|  | CDI | 22.73 (9.29), n = 11 | 16.29 (10.24), n = 7 | - |  |
|  | FQoLS^Q^ | 17.14 (6.04), n = 11 | 21.29 (7.68) n = 7 | - | Significant increase in QoL was found by the conclusion of BA (M = 21.29 [7.68]: Z = −2.21, *p*=.03). |
| Ritschel et al (2016) [43] | CDRS-R | 60.29 (10.24), n=28 | *NR,* n=22 | *NR*, n=16 | Significant decreases in scores from baseline to end of treatment on the CDRS-R, *F*(2, 40) = 33.60, *p*< .001, g2 *p*=0.63, and BDI-II, *F*(2, 40) = 34.14, *p* < .001, g2 *p*=0.63 and the CBCL (*t*(20)=5.67, *p*<.001, *d*=1.24. |
|  | CBCL withdrawn/depressed scale | 72.25 (9.31), n=28 | *NR,* n=22 | *NR,* n=16 |  |
|  | BDI-II | 27.84 (10.69), n=28 | *NR,* n=22 | *NR*, n=16 |  |
| Ritschel et al. (2011) [49] | CDRS-R | P1:50  P2:46  P3:53  P4:74  P5: 54  P6: 69  *M*=57.67(11.18), n=6 | P1: -  P2: 30  P3: 21  P4: 24  P5: 24  P6: 24  *M*=27.67(8.07), n=5 | - | Depression scores decreased significantly on both the CDRS, *F*(1,5) = 19.94, *p*<.01, and the BDI-II, *F*(1,5) = 330.00, *p<*.001. |
|  | BDI-II | P1:33  P2: 37  P3: 18  P4: 27  P5: 27  P6: 26  *M*=28.00(6.51), n=6 | P1: -  P2: 14  P3: 1  P4: 3  P5: 2  P6: 3  *M*= 6.00(5.87), n=5 | - |  |
| Ruggiero et al (2005) [38] | BDI | 13, n=1 | 2, n=1 |  | Meaningful reduction in depression scores from pre-to-post-treatment |
| Shadan et al. (2021) [50] | MFQ-P | 39, n=1 | 4 weeks after starting treatment: 21, n=1  6 weeks after starting treatment: 13, n=1 | - | Reductions in both depression and anxiety during treatment completion. Participant no longer meeting clinical depression criteria at 6-weeks follow-up. (MFQ-P score for follow-up not reported) |
|  | SCARED-P^A^ | 24, n=1 | 4 weeks after starting treatment: 15, n=1  6 weeks after starting treatment: 10, n=1 | - |  |
| Wallis et al. (2012) [51] | CES-D | *NR* | - | - | All participants (n=5) had reduced depressive symptoms between pre-and-post-treatment. |
|  | BDI-II | P1: 44^⤉^  P2: 19^⤉^  P3: 21^⤉^  P4: 22^⤉^  P5: 24^⤉^  *M=*26(9.14),n=5 | P1: 17^⤉^  P2: 2^⤉^  P3: 7^⤉^  P4: 17^⤉^  P5: 18^⤉^  *M=* 12.2(6.49), n=5 | - |  |
| Weersing et al. (2008) [52] | CDI-P | P1:26^⤉^  P2:40^⤉^ | P1:5^⤉^  P2:0^⤉^ | P1:5^⤉^  P2:5^⤉^ | Reductions in depression scores for both participants from baseline to follow-up. However, for one participant depression was seen to increase at 6 months follow-up on the parent-reported measure. |
|  | CDI-C | P1:25^⤉^  P2:21^⤉^ | P1:11^⤉^  P2:7^⤉^ | P1:4^⤉^  P2:0^⤉^ |  |
|  | SCARED-P^A^ | P1:38^⤉^  P2:26^⤉^ | P1:9  P2:7 | P1:8^⤉^  P2:16^⤉^ | Reductions in anxiety scores for both participants from baseline to follow-up on the child-reported measure. However, for one participant anxiety was seen to increase at 6 months follow-up on the parent-reported measure. |
|  | SCARED-C^A^ | P1:44^⤉^  P2: 21^⤉^ | P1:23^⤉^  P2: 3^⤉^ | P1:18^⤉^  P2: 0^⤉^ |  |

**Notes.** *Final follow-up completed. *NR*=Not reported. ^A^Anxiety measures, ^Q^QoL measures. *UC:* Usual Care;** Only raw scores provided, ^⤉^Scores not presented as numerical values, presented in a line graph and interpreted by review authors.

**S5.** Funnel Plot of studies using CDRS-R

**S6.** Funnel plot of studies using standardised mean difference

**S7. Further information about QOL.**

Three studies [34, 40, 42] assessed QoL. Although Douleh [40] reported values relating to Health-Related QoL (HRQoL), the measure used was not reported. Grudin et al. [34] administered the KIDSCREEN-10 and found significant improvements on both child and parent-reported HRQoL scores across all groups from baseline to 3-months follow-up: Guided BA (child-reported: *B=*3.03, *p<*0.001, 95%CI 1.80 to 4.27; parent-reported: *B=*2.16, *p<*0.001, 95%CI 1.14 to 3.17), self-guided BA (child-reported: *B=*3.46, *p<*0.001, 95%CI 1.93 to 4.99; parent-reported: *B=*2.25, *p<*0.01, 95%CI 0.74 to 3.76), usual care (child-reported: *B=*2.71, *p<*0.05, 95%CI 0.65 to 4.76; parent-reported: *B=*1.82, *p<0.05*, 95%CI 0.01 to 3.62). Riley and Gaynor [42] examined BA and QoL using the FQOLS, reporting a significant increase in QoL by the conclusion of BA (*M* = 21.29 [7.68]: *Z* = -2.21, *p*=.03).

**S8. Further information about online BA deliveries.**

Two studies delivered BA adopting an online format. In Schleider et al. [23] participants independently completed a single, 30-minute BA session delivered entirely online. The participants found the intervention acceptable, and depression improved between pre-treatment and 3-months follow-up. Furthermore, those randomised to BA were more likely to complete the programme they received. The authors concluded that the programme’s brevity and flexibility may enable provision of support to some young people who might not otherwise access help at all. Nevertheless, they also observed that single session, online interventions like this would be better complementing, rather than replacing, other forms of routine care.

In Grudin et al. [34] participants were randomised to an internet-based BA, either with or without therapist support, or usual care. BA was delivered in eight chapters over up to 10-weeks in 30–60-minute sessions. Where the BA was delivered alongside therapist support, professionals allocated time to contact young people within the online programme and scheduled additional telephone calls where necessary. Treatment completion was higher with therapist support: 70% of participants completed all eight chapters compared to 30% in the self-guided group. In both BA groups there was a significant reduction in depression symptoms (but not for usual care) and young people found the BA acceptable.

Although the BA in Dubicka et al [41] was developed for in-person delivery, the COVID-19 pandemic forced some sessions to be conducted online. This was distinct to Schleider et al. [23] and Grudin et al. [34] where BA was delivered through computer prompts and, where a therapist was involved, this was to advise the young person on completing these prompts. The Dubicka et al. [41] study demonstrated that BA could be successfully delivered directly by therapists in an online rather than physically present format.

As only three studies incorporated online/telephone facilitated BA, we could not carry out any subgroup analyses; however, the studies’ collective findings support online and telephone-facilitated BA as an acceptable and effective intervention.
